# The herpevac trial for women: Sequence analysis of glycoproteins from viruses obtained from infected subjects

**DOI:** 10.1371/journal.pone.0176687

**Published:** 2017-04-27

**Authors:** Miguel A. Minaya, Maria Korom, Hong Wang, Robert B. Belshe, Lynda A. Morrison

**Affiliations:** 1 Department of Molecular Microbiology and Immunology, Saint Louis University School of Medicine, St. Louis, Missouri, United States of America; 2 Department of Internal Medicine, Saint Louis University School of Medicine, St. Louis, Missouri, United States of America; University of California Irvine Medical Center, UNITED STATES

## Abstract

The Herpevac Trial for Women revealed that three dose HSV-2 gD vaccine was 58% protective against culture-positive HSV-1 genital disease, but it was not protective against HSV-2 infection or disease. To determine whether vaccine-induced immune responses had selected for a particular gD sequence in strains infecting vaccine recipients compared with viruses infecting control subjects, genetic sequencing studies were carried out on viruses isolated from subjects infected with HSV-1 or HSV-2. We identified naturally occurring variants among the gD sequences obtained from 83 infected subjects. Unique or low frequency amino acid substitutions in the ectodomain of gD were found in 6 of 39 HSV-1-infected subjects and in 7 of 44 HSV-2-infected subjects. However, no consistent amino acid change was identified in isolates from gD-2 vaccine recipients compared with infected placebo recipients. gC and gE surround and partially shield gD from neutralizing antibody, and gB also participates closely in the viral entry process. Therefore, these genes were sequenced from a number of isolates to assess whether sequence variation may alter protein conformation and influence the virus strain’s capacity to be neutralized by vaccine-induced antibody. gC and gE genes sequenced from HSV-1-infected subjects showed more variability than their HSV-2 counterparts. The gB sequences of HSV-1 oral isolates resembled each other more than they did gB sequences rom genital isolates. Overall, however, comparison of glycoprotein sequences of viral isolates obtained from infected subjects did not reveal any singular selective pressure on the viral cell attachment protein or surrounding glycoproteins due to administration of gD-2 vaccine.

## Introduction

Herpes simplex virus 1 (HSV-1) and HSV-2 are highly related human herpesviruses. Their 152 to 155 kb colinear genomes share 87% amino acid sequence identity and encode 84 proteins [1]. Both viruses also share structural features including an icosahedral capsid, a dense layer of tegument proteins, and a host cell-derived lipid envelope studded with viral glycoproteins important in cell attachment and penetration. Historically HSV-1 caused most oral infections and HSV-2 most genital infections; however, HSV-1 is now responsible for a majority of genital infections [2–7]. Over 400 million people world-wide are thought to have genital HSV infections [8]. In addition to the direct impacts of HSV on the physical and psychosocial health of infected individuals, women can pass the virus to their babies during birth, resulting in severe and often lethal disease [9]. Previous infection with HSV also increases the risk of co-infection with HIV [10,11], and infectious HIV is shed from HSV-2 genital ulcers [12], making HIV transmission more likely [13]. The ability to control HSV infections would have a wide-ranging positive impact on public health.

The lifecycle of HSV-1 and HSV-2 alternates between lytic infectious and latent phases. The viruses typically enter the body through mucosal epithelium or abraded skin. Replication in epithelial cells leads to lysis which rapidly puts virus in contact with nerve termini innervating the site of infection. Intra-axonal transport conveys the virus to nerve cell bodies in sensory ganglia. Here the virus establishes a latent infection that persists for the lifetime of the infected individual. Periods of viral reactivation permit recurrent virus shedding in the periphery and re-infection of the epithelium, thus perpetuating the lytic-latent cycle and providing an opportunity for transmission. Nine viral glycoproteins play significant and in some cases essential roles in the virus lifecycle. Glycoprotein D (gD) interacts with the cellular receptors nectin 1, HVEM, and also nectin 2 in the case of HSV-2 [14]. Conformational changes triggered in gD by receptor binding lead to interaction of gD with gH/gL [15]. Subsequent interaction of activated gH/gL with gB stimulates gB fusogenic activity [16–18]. Thus, gD binding initiates several interactions critical for successful HSV infection.

Glycoproteins gC and gE play strategic roles in HSV immune evasion. gC binds the C3b component of complement to prevent complement activation and virolysis [19,20]. gE in complex with gI acts as an immunoglobin Fc receptor, preventing antibody-mediated viral neutralization [21–23], and facilitating clearance of viral antigens and antiviral antibody from the cell surface [24]. gC and gE also surround and partially shield gD from neutralizing antibody attack that could interfere with virus entry [25].

HSV-1 and HSV-2 have a relatively low mutation rate due to the proofreading activity of their DNA polymerases [1]. Nonetheless, sequence diversity has been noted in certain glycoproteins [26–29]. Considered on a global scale, HSV glycoprotein sequence diversity increases with geographic distance [28]. Development of a vaccine that can effectively counter this breadth of diversity among strains is a significant challenge. Attempts to prevent HSV infection to date have focused primarily on the use of viral glycoprotein subunit vaccines. A vaccine composed of gD adjuvanted with alum and 3-*O*-deacylated monophosphoryl lipid A (ASO4) showed promise in early vaccine trials [30], prompting a large, multicenter Phase III trial, the Herpevac Trial for Women [31]. A total of 8,323 young adult women who were seronegative for both HSV-1 and HSV-2 received three doses of the ectodomain (amino acids 26–309) of HSV-2 gD in adjuvant or a control hepatitis A virus (HAV) vaccine. The gD-2 vaccine provided 58% protection against HSV-1 culture positive disease but did not protect against HSV-2. How HSV-2 successfully evaded the vaccine-induced immune response has been a central question in understanding the outcome of the trial.

Results from the Herpevac Trial for Women indicated protection was associated with antibody titer but not CD4 or CD8 T cells against HSV-1, and therefore neutralizing antibodies evoked by vaccination have been considered critical to successfully preventing HSV infection [32]. Whether neutralizing antibodies among the antibodies measured by ELISA positively correlate with protection is the subject of an ongoing study. Mechanisms of virus neutralization could involve blocking gD’s nectin-1 or HVEM binding domains [33–38], or preventing gD association with gH/gL [37]. Antibody escape variants have been noted for many viruses. Thus, vaccine-induced antibody responses could limit infection to particular strains whose glycoprotein sequences facilitate immune evasion. Pre-existing antibody may also be a driving force for selection of a variant with increased fitness within a vaccinated, infected individual. We therefore determined whether glycoprotein sequences differed between virus isolates from gD-2 vaccine recipients in the Herpevac Trial who became infected and isolates from infected, control-vaccinated subjects.

## Materials and methods

### Cells and viruses

Vero (African green monkey kidney) cells were originally acquired from the laboratory of David Knipe and were maintained in Dulbecco’s modified Eagle's medium (DMEM) supplemented with 3% newborn calf and 3% bovine growth sera, 100 IU/ml penicillin and 0.1 mg/ml streptomycin (1x P/S). HSV-1 and HSV-2 swab isolates in transport medium collected during the Herpevac Trial for Women were thawed and 100 μl were inoculated onto Vero cell monolayers in T75 flasks. Monolayers were incubated until cytopathic effect reached 100%, and cell lysate stocks of the isolates were prepared as previously described [39]. The study was approved by the Saint Louis University Institutional Review Board (IRB number 24706) and subjects provided written consent to future use of their samples.

### DNA isolation and sequencing

Viral DNA was purified from a portion of each virus lysate using QIAmp DNA Mini kit (Qiagen, Valencia, CA) according to the manufacturer’s recommendations. Glycoprotein genes were PCR-amplified using strain-specific primers (Table 1). Amplification reactions used a reaction mixture containing 0.75 μl of forward and reverse primer (10 mM), 2.5 μl 10X AccuPrime^TM^ *Pfx* Reaction Mix (Invitrogen), 2.5 μl Betaine solution (5M) (Sigma), 2 μl MgCl_2_ (50mM), 1.5 μl DMSO, 0.5 μl *Taq* DNA Polymerase, 2 to 4 μl of template DNA in a total reaction vol of 25 μl. The amplification parameters consisted of an initial denaturing step of 2 min at 95°C, followed by 39 cycles of 20 sec denaturing at 95°C, 30 sec annealing at primer-specific temperature, and 3 min extension at 68°C, followed by a final extension step of 5 min at 68°C. DNA products were purified by agarose gel electrophoresis and extracted using a PureLink Quick Gel Extraction kit (Invitrogen, Grand Island, NY). Sanger sequencing of purified PCR products was conducted by GeneWiz, Inc. (South Plainfield, NJ).

**Table 1:** Primers used for amplification of HSVgB, gC, gD, and gE genes.

Glycoprotein	Name	Genome location^a^	Sequence 5’ to 3’
HSV-1 gB	gB1-FWD	52616	CGCCGATTTGTTCGTCTC
	gB1-REV	56125	CGTCAGCGAATTTCAGAG
HSV-1 gC	gC1-FWD	95768	TCCGAGGTTGTCGTGTATG
	gC1-REV	98149	GTTGAACTCGTGCCATCCGCTGTC
HSV-1 gD	gD1-FWD	138074	GCCGTGTGACATTATCGTCCATAC
	gD1-REV	139758	CGGCGTCCACAAATGAGTTTGATACCAG
HSV-1 gE	gE1-FWD	140831	ACCGCAGTCACTGAGTTG
	gE1-REV	143263	CGCTTTCCGAGTAGTAGG
HSV-2 gB	gB2-FWD	53108	ACTCGCCCGCTTCATCATGG
	gB2-REV	56589	GGGAAAGTGGGCCGAATGAC
HSV-2 gC	gC2-FWD	96855	AGGCGGGCCCATCACTGTTAGGGTGTTA
	gC2-REV	98691	GCCATAGCACCACCGCGGGCTTGAATAT
HSV-2 gD	gD2-FWD	140944	GTGTGGTGTTCGGTCATAAG
	gD2-REV	142270	TCCAAGTCCCACCCAATTAC
HSV-2 gE	gE2-FWD	143400	GTCCACGACCATGCCTTCCCTAAC
	gE2-REV	145681	ACGGCGAGCCAGGTGACGAATTG

^a^ Nucleotide position in the complete genome of HSV-1 (GenBank accession #JQ673480) or HSV-2 (JN561323).

### Sequence analyses

As a quality control measure, each chromatogram was visually inspected for miscalled nucleotides and overlapping peaks that could indicate a mixed population. Complementary strands were assembled and verified using Clone Manager 9 Professional Edition. The obtained sequences were aligned and adjusted manually using *MEGA* (v7.0.14) [40]. Nucleic acid sequences of glycoproteins from HSV-1 isolates were aligned to reference sequences from HSV-1 strain KOS (Table 2) [41]. Nucleic acid sequences containing gD coding regions of HSV-2 isolates were aligned to reference sequences from HSV-2 strain G [42] or SD90e [43]. SD90e furnished the reference sequences for the remaining glycoproteins. gD sequences from HSV-1 strains F [42], 17 [44], and McKrae [45], and HSV-2 strains 186 [46] and 333 [47] were also included in some comparisons. The percentage of polymorphic nucleotides and pairwise comparison to the reference sequence [transition/transversion (T_S_/T_V_) ratio] for each glycoprotein (gB, gC, gD and gE) of HSV-1 and HSV-2 strains were assessed using PAUP* *4*.*0 beta10* [48]. The collection of isolates in this study was compared with verified primary clinical isolates previously deposited in GenBank. Because of the low numbers of polymorphisms per sequence, the Ts/Tv ratio is expressed as the sum of the transitions across the isolates divided by the sum of the transversions. GenBank accession numbers for all the glycoprotein sequences obtained herein and previously sequenced corresponding genes of primary isolates are listed in S1 Table. Only nucleotides encompassing the ORF of each protein were considered, excluding INDELs [49]. Two groups of strains were used: the newly sequenced strains presented in this research, and verified low passage clinical strains previously uploaded to GenBank (S1 Table) [50]. Variation of nucleotides across the alignment was calculated using the HSV-1 KOS reference strain for all HSV-1 isolates, the HSV-2 strain G for gD of HSV-2 samples, and the HSV-2 strain SD90e for HSV-2 gB, gC and gE.

**Table 2:** Number of subjects (isolates).

Infection		Vaccine		
		HSV	HAV	
**HSV-1**	Oral	6 (7)	3 (3)	
	Genital	12 (13)	16 (18)	
	Rectal		2 (2)	
	Total	18 (20)	21 (23)	39 (43)
**HSV-2**	Oral	0 (2)		
	Genital	29 (35)	12 (16)	
	Rectal	2 (3)		
	Buttock	1 (1)		
	Total	32 (41)	12(16)	44 (57)
				83 (100)

The frequencies of non-synonymous (*d*N), and synonymous (*d*S) substitutions were calculated based on the codon-aligned nucleotide sequences in a data set that included all gD sequences from 39 HSV-1 infected subjects and 44 HSV-2 infected subjects. *d*N/*d*S ratios were calculated for HSV-1 and HSV-2 gB, gC and gE based on sequences determined for a subset of isolates. These calculations were performed using the *SNAP* (*Synonymous Non-synonymous Analysis Program v2*.*1*.*1*) website [51], which determines the number of non-synonymous v. synonymous base substitutions for all pairwise comparisons of sequences in an alignment. To investigate positive selection on a site-by-site basis we used an agreement-based inference that included five methods: Fixed Effects Likelihood (FEL), Internal Fixed Effects Likelihood (IFEL), Single-Likelihood Ancestor Counting (SLAC), Mixed Effects Model of Evolution (MEME), and Fast Unbiased Bayesian Approximation (FUBAR) (*DataMonkey* software package; [52,53]). Following the criteria of Lamers *et al*. [28], positive selection was considered likely when at least three of these methods indicated positive selection at a particular coding position.

The SLAC method, a substantially modified and improved derivative of the Suzuki-Gojobori method [54], involves counting the number of *d*N and *d*S changes and testing whether *d*N is significantly different from *d*S. The FEL method [55] incorporates models of nucleotide substitution bias and variation in both non-synonymous and synonymous substitution and thus estimates the *d*N and *d*S rates at each site. The IFEL method [56] infers whether the instantaneous *d*S site rate is lower than the instantaneous *d*N site rate. IFEL differs in that it is used to determine whether selection is occurring at the population level by investigating sitewise selection on internal branches of a phylogenetic tree [56]. The MEME method [57] detects adaptive evolution and can identify instances of positive selection affecting individual codon positions. Finally, the FUBAR method [58] detects positively selected codon positions using a Bayesian approximation. A Markov Chain Monte Carlo routine used in this method allows flexible prior specification with no parametric constraints on the prior shape, and visualizes Bayesian inference for each nucleotide. The cutoff P value used in FEL, IFEL, SLAC and MEME was 0.1, while the value used in FUBAR was 0.9 as recommended in *DataMonkey*.

### Statistical analyses

The proportion of sequences from gD-2 vaccine versus control vaccine recipients which contained ectodomain polymorphisms was compared using the Fisher exact test. Statistical significance of the *d*N/*d*S ratios of sequences from gD-2 vaccine recipients and control vaccine recipients was determined by an unpaired *t* test.

## Results

Genetic sequencing studies were carried out on gD of viruses isolated from women who became infected with HSV-1 or HSV-2 during the trial to establish whether amino acid variants of the cell attachment protein correlated with successful infection. Subjects had received up to three doses of either HSV gD-2 vaccine in adjuvant or HAV vaccine as a control vaccine. A total of 100 primary or recurrent isolates were obtained from 39 subjects infected with HSV-1 and 44 subjects infected with HSV-2 (Table 2). Of the 39 HSV-1-infected subjects, 30 (77%) had genital (or rectal) infections and 9 (23%) had oral infections. A larger proportion of the culture-positive HSV-1 genital infections occurred in subjects receiving control vaccine than gD-2 vaccine [18 (2 were rectal) v. 12 subjects; 60% v. 40%]. Nearly all culture-positive infections with HSV-2 occurred in the genital or rectal mucosa, 12 in control vaccine recipients and 31 in gD-2 vaccine recipients. One of the gD-2 vaccine recipients acquired a buttock infection with HSV-2, and two experienced oral as well as genital infections. Isolates obtained from subjects with recurrent disease in the months after primary infection were also sequenced.

### gD sequences

**Fig 1:**
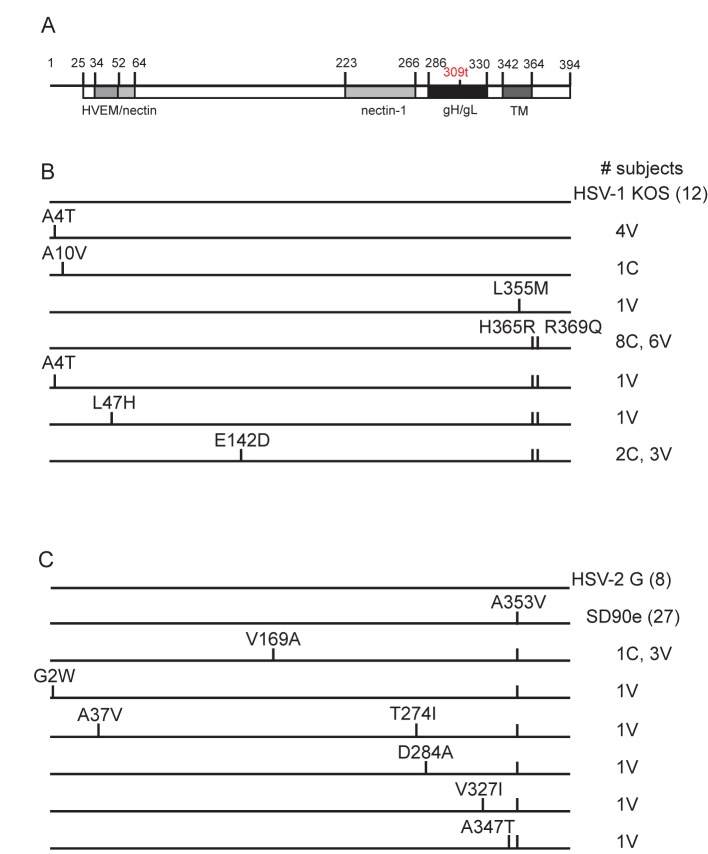
gD amino acid sequences of isolates from the Herpevac Trial. The U_S_6 gene of isolates from subjects infected during the Herpevac Trial was sequenced and then translated. A) Diagram of the gD protein showing boundaries of functional domains; TM, transmembrane. 309t in red indicates the site of truncation in the HSV-2 gD vaccine construct. Substitutions in the gD amino acid sequences of isolates from B) 39 HSV-1-infected subjects compared with reference sequence KOS (HSV-1 laboratory strain), and C) 44 HSV-2-infected subjects compared with G (HSV-2 laboratory strain and derivation of the gD-2 vaccine), and SD90e (HSV-2 primary isolate from South Africa) are shown. Numbers to the right of each sequence representation indicate the number of subjects sharing the amino acid sequence. C, control vaccinated subject; V, gD-2 vaccinated subject.

Forty-three HSV-1 gD gene sequences were determined for primary or recurrent isolates from the 39 HSV-1-infected subjects, and were compared with gD from HSV-1 strain KOS as a reference sequence. Ten of the 39 subjects’ gD sequences were identical to HSV-1 KOS even at the nucleic acid level, and 14 at the amino acid level. Nucleotide polymorphisms in other gD sequences were scattered throughout the open reading frame, but only 7 non-synonymous changes were observed (Fig 1B). Two of these, A4T and A10V, lie within the leader sequence cleaved to form the mature protein. One amino acid sequence variant within the ectodomain may represent a naturally occurring polymorphism. Specifically, an E142D substitution in 5 subjects’ gD sequence also appeared in a patient isolate in GenBank, E03. Notably, two unique amino acid changes were also observed: L47H was found in one gD-2 vaccine recipient, and L355M in the transmembrane domain was found in another gD-2 vaccine recipient. Twenty of the 39 subjects had H365R and R369Q substitutions in the cytoplasmic domain that are also present in laboratory strains F, 17 and McKrae. Interestingly, one subject’s isolate contained both the A4T and H365R/R369Q substitutions, suggesting a possible recombination event. None of these polymorphisms was associated with a particular route of infection. In all gD sequences bearing nucleotide polymorphisms, the same nucleotide was almost always substituted at a given position. The largest number of nucleotide changes per gD sequence was 11, with an average of 3.7 per subject (0.33%). These resulted in 3 or fewer amino acid substitutions per subject, with an average of only 0.23 non-synonymous changes per subject in the region spanned by the vaccine.

Fifty-seven gD gene sequences were determined for primary or recurrent isolates from the 44 HSV-2-infected subjects, then were compared with gD from HSV-2 strain G (from which the vaccine was derived) as a reference sequence. The gD sequence of two other HSV-2 laboratory strains, 333 and 186, and the field isolate SD90e were also compared. Only eight non-synonymous amino acid substitutions were observed among the gD sequences obtained from HSV-2-infected subjects (Fig 1C). One of these changes, G2W, occurred in the leader sequence. Isolates from 4 other HSV-2-infected subjects contained unique amino acid change(s) in the gD ectodomain: A37V and T274I (occurring together), D284A, or V327I. In addition, 4 of the 44 subjects had a V169A substitution which was also found in a gD sequence previously submitted to GenBank, Pt10, suggesting that it is a naturally occurring polymorphism. A347T and A353V substitutions were found in the transmembrane domain of gD. The former was unique, whereas 39 of 44 isolates had the A353V substitution which was also present in the laboratory strains 186 and 333. The largest number of nucleotide changes in an HSV-2 gD sequence was 5, with an average of only 1.16 synonymous substitutions and 1.09 non-synonymous substitutions per subject (total = 0.18%). These resulted in 3 or fewer amino acid substitutions in the gD sequence of a given subject, and within the region spanned by the vaccine an average of only 0.21 non-synonymous changes occurred per subject. When oral and genital infections with HSV-2 occurred within the same individual, the gD sequences were identical.

In summary, 7 infrequent polymorphisms were found in the gD ectodomain of isolates from 13 subjects, 6 subjects infected with HSV-1 and 7 with HSV-2. The two changes in HSV-1-infected subjects (L47H and E142D) and 4 of the 5 in HSV-2-infected subjects (A37V, V169A, T274I and D284A) occurred in the portion of the ectodomain spanned by the vaccine. Ten gD-2-vaccinated subjects became infected with viruses that had ectodomain polymorphisms compared with only 3 of the control vaccine recipients, but this difference was not statistically significant (P = 0.222). None of the gD sequences from 4 recurrent isolates obtained from among the 39 HSV-1-infected subjects and 13 recurrent isolates from among the 44 HSV-2-infected subjects differed from the subject’s primary isolate at the amino acid or nucleotide levels, consistent with another recent report [59].

### Sequences of glycoproteins C and E

**Fig 2:**
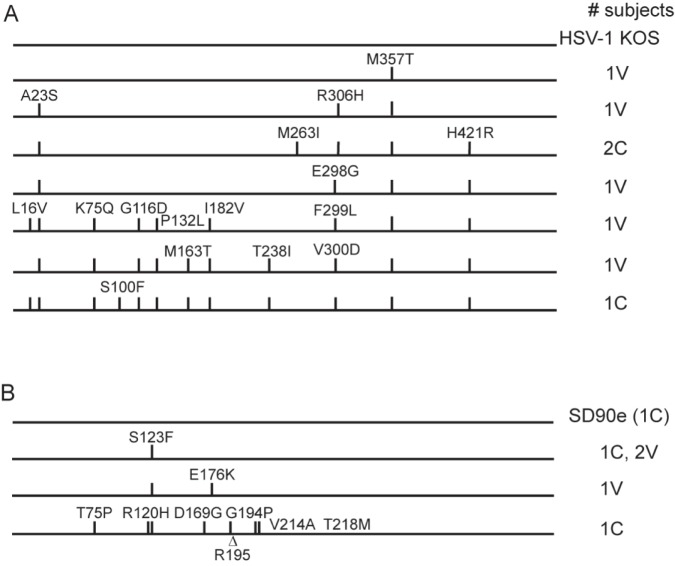
gC amino acid sequences of isolates from the Herpevac Trial. The UL44 gene encoding gC was sequenced from selected subjects’ isolates and aligned to reference sequences, then translated. Amino acid substitutions are diagramed for A) gC from HSV-1-infected subjects, B) gC from HSV-2-infected subjects. Numbers to the right of each sequence representation indicate the number of subjects sharing the amino acid sequence. C, control vaccinated subject; V, gD-2 vaccinated subject. The triangle represents a deletion of one amino acid.

gC and gE surround and partially shield gD from neutralizing antibodies that could interfere with gD receptor interaction or its association with gH/gL [25]. We therefore sequenced these genes from several isolates to determine whether sequence variation may alter protein conformation and influence the virus strain’s capacity to be neutralized by vaccine-induced antibody. Eight HSV-1 gC genes were sequenced from subjects’ isolates, half from gD-2 vaccine recipients and half from those receiving control vaccine. Five gC amino acid sequences closely resembled the reference strain KOS (Fig 2A). The last three HSV-1 gC sequences contained numerous amino acid substitutions in a similar pattern, primarily in the N-terminal third of the molecule. Nucleotide changes were more numerous overall than with HSV-1 gD, ranging from 1 to 19 per sequence (average 11.1, 0.7%). Of six HSV-2 gC genes sequenced, 5 contained only two amino acid substitutions compared with the field isolate SD90e (Fig 2B). Divergence at the nucleotide level was also very low for 5 of 6 isolates, with 0 to 4 differences (average 2, 0.14%). However, the sixth HSV-2 gC sequence contained 8 amino acid substitutions relative to gC of SD90e, and 11 nucleotide substitutions (divergence of 0.76%). Overall HSV-2 gC nucleotide divergence was thus 0.21%.

**Fig 3:**
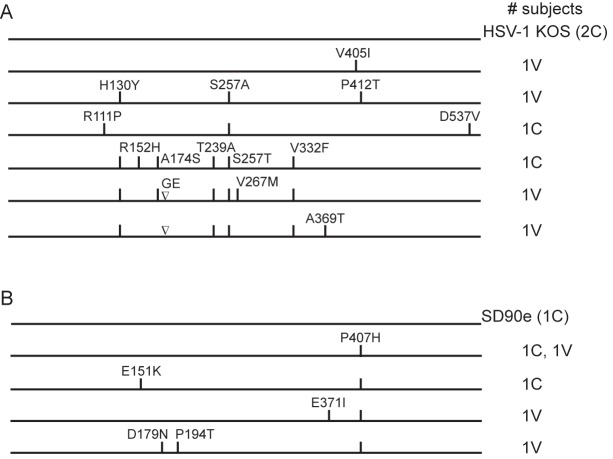
gE amino acid sequences of isolates from the Herpevac Trial. The US8 gene encoding gE was sequenced from selected subjects’ isolates and aligned to reference sequences, then translated. Amino acid substitutions are diagramed for A) gE from HSV-1-infected subjects, and B) gE from HSV-2-infected subjects from the same sets of subjects as described in Fig 2. Numbers to the right of each sequence representation indicate the number of subjects sharing the amino acid sequence. C, control vaccinated subject; V, gD-2 vaccinated subject. The inverted triangle represents an insertion of two amino acids (GE).

gE sequences were also determined for the same subset of HSV-1 and HSV-2 isolates. Two HSV-1 gE amino acid sequences were identical to KOS, and three more were very similar to KOS (Fig 3A). The other three gE amino acid sequences resembled each other, but differed substantially from KOS. At the nucleotide level, the number of substitutions ranged from 1 to 27 (average 0.55%). Interestingly, the three HSV-1 isolates with gE amino acid sequences most divergent from KOS also had the most divergent gC sequences. In contrast to HSV-1 gE, the six HSV-2 gE sequences had very few polymorphic residues compared with gE of SD90e (Fig 3B). Nucleic acid substitutions were also rare, ranging from 0 to 3 (average 0.12%). No correlations existed between gC or gE sequences and subjects’ route of infection or receipt of gD-2 vaccine (data not shown).

### gB sequences

**Fig 4:**
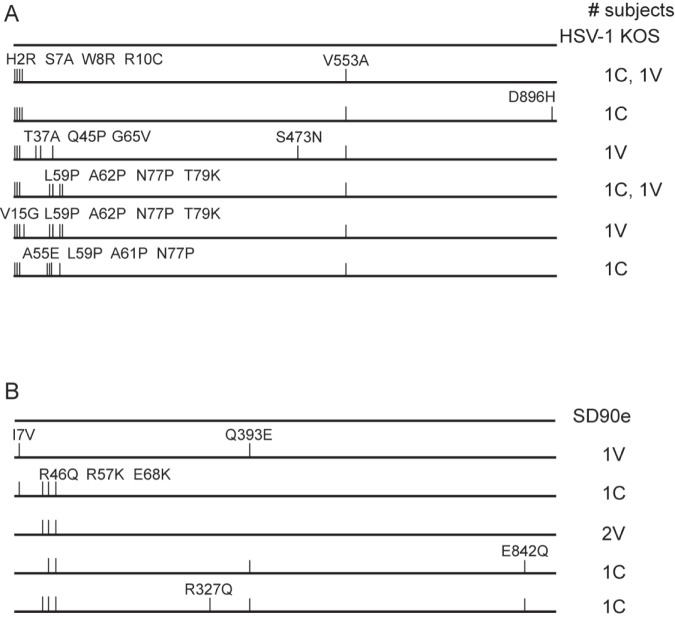
gB amino sequences. The UL27 gene encoding gB was sequenced from selected subjects’ isolates and aligned to reference sequences. Amino acid substitutions in the translated sequence are diagramed for gB from A) HSV-1-infected subjects, and B) HSV-2-infected subjects from the same sets of subjects as described in Fig 2. Numbers to the right of each sequence representation indicate the number of subjects sharing the amino acid sequence. C, control vaccinated subject; V, gD-2 vaccinated subject.

Because gB and gD each interact with the gH/gL complex during the entry process [60], conformational changes accompanying gB sequence alterations could conceivably influence the capacity of vaccine-induced antibody to access gD. We therefore sequenced the gB genes of the same subset of 8 HSV-1 and 6 HSV-2 isolates. Focusing on the ectodomain of the mature gB protein, 3 gB amino acid sequences of HSV-1 genital tract isolates had a V553A substitution but were otherwise identical to the KOS reference sequence (Fig 4A). An additional sequence bore 3 substitutions in the N-terminus and a unique S473N substitution. Nearly all polymorphic residues in the remaining 4 isolates were located within the N-terminal 79 amino acids of gB; notably, several of the substitutions were to proline residues and all 4 were oral isolates. All nucleotide sequences of gB from HSV-1 isolates, however, contained numerous (17 to 22) nucleotide changes in the ORF compared with KOS (average 0.70%). HSV-2 gB amino acid sequences from 5 out of 6 subjects also varied primarily in the same N-terminal portion as the HSV-1 isolates (Fig 4B), with an average of 3 substitutions per subject. Interestingly, most HSV-2 gB nucleotide changes were non-synonymous; however, no correlation between pattern of amino acid substitutions and vaccination with gD-2 or control vaccine was observed. HSV-2 gB nucleotide differences relative to SD90e ranged from 4 to 7 per sequence (average 0.20%). Table 3 summarizes the nucleotide variation among glycoprotein sequences obtained. The rate of polymorphisms when compared with sequences already deposited in GenBank was similar for all glycoproteins except for HSV-1 gD, whose sequences were less polymorphic in our study. The transition/transversion (T_S_/T_V_) ratio, however, was lower for HSV-1 gD in our study than previously deposited sequences (Table 3), indicating a greater proportion of the polymorphisms were transversions. Overall, the lowest (T_S_/T_V_) ratios occurred in HSV-2 gC and gE.

**Table 3:** Nucleotide sequence diversity in glycoproteins B, C, D and E among HSV-1 and HSV-2 primary isolates.

	**HSV-1**							
**Glycoprotein sequences**	**gB**		**gC**		**gD**		**gE**	
	**Newly sequenced**	**GenBank**	**Newly sequenced**	**GenBank**	**Newly sequenced**	**GenBank**	**Newly sequenced**	**GenBank**
**Number of sequences**^**a**^	8	22	8	72	39	24	8	27
**% polymorphic nt.**^**b**^	0.70%	0.80%	0.70%	0.80%	0.33%	0.60%	0.55%	0.34%
**T**_**S**_ **/ T**_**V**_ **ratio**^**c**^	1.88	2.44	2.54	1.14	2.28	2.30	2.36	1.12
**Avg. G+C content**	66.46%	66.47%	68.20%	68.19%	64.62%	64.70%	66.42%	66.62%
	**HSV-2**							
**Glycoprotein sequences**	**gB**		**gC**		**gD**		**gE**	
	**Newly sequenced**	**GenBank**	**Newly sequenced**	**GenBank**	**Newly sequenced**	**GenBank**	**Newly sequenced**	**GenBank**
**Number of sequences**	6	107	6	5	44	49	6	41
**% polymorphic nt.**	0.20%	0.30%	0.21%	0.19%	0.18%	0.24%	0.12%	0.15%
**T**_**S**_ **/ T**_**V**_ **ratio**	2.11	1.76	8.06	13.00	11.33	13.50	0.72	0.55
**Avg. G+C content**	67.06%	67.09%	70.56%	70.53%	64.32%	64.27%	68.53%	68.54%

^**a**^ Number of verified glycoprotein sequences used in these comparisons of Herpevac Trial isolates and existing GenBank entries (S1 Table). Only nucleotides encompassing the ORF of each protein were considered.

^**b**^ Average percentage of nucleotides (nt.) differing from HSV-1 reference sequence KOS (JQ673480) or HSV-2 reference strains G (KY933650) for gD or SD90e (JN561323) for the other glycoproteins. Positions in INDELs were excluded.

^**c**^ Average Transition (T_S_) / Transversion (T_V_) ratio when compared with the HSV-1 reference sequence KOS or the HSV-2 reference strains G for gD or SD90e for the other glycoproteins. Positions in INDELs were excluded.

**Fig 5:**
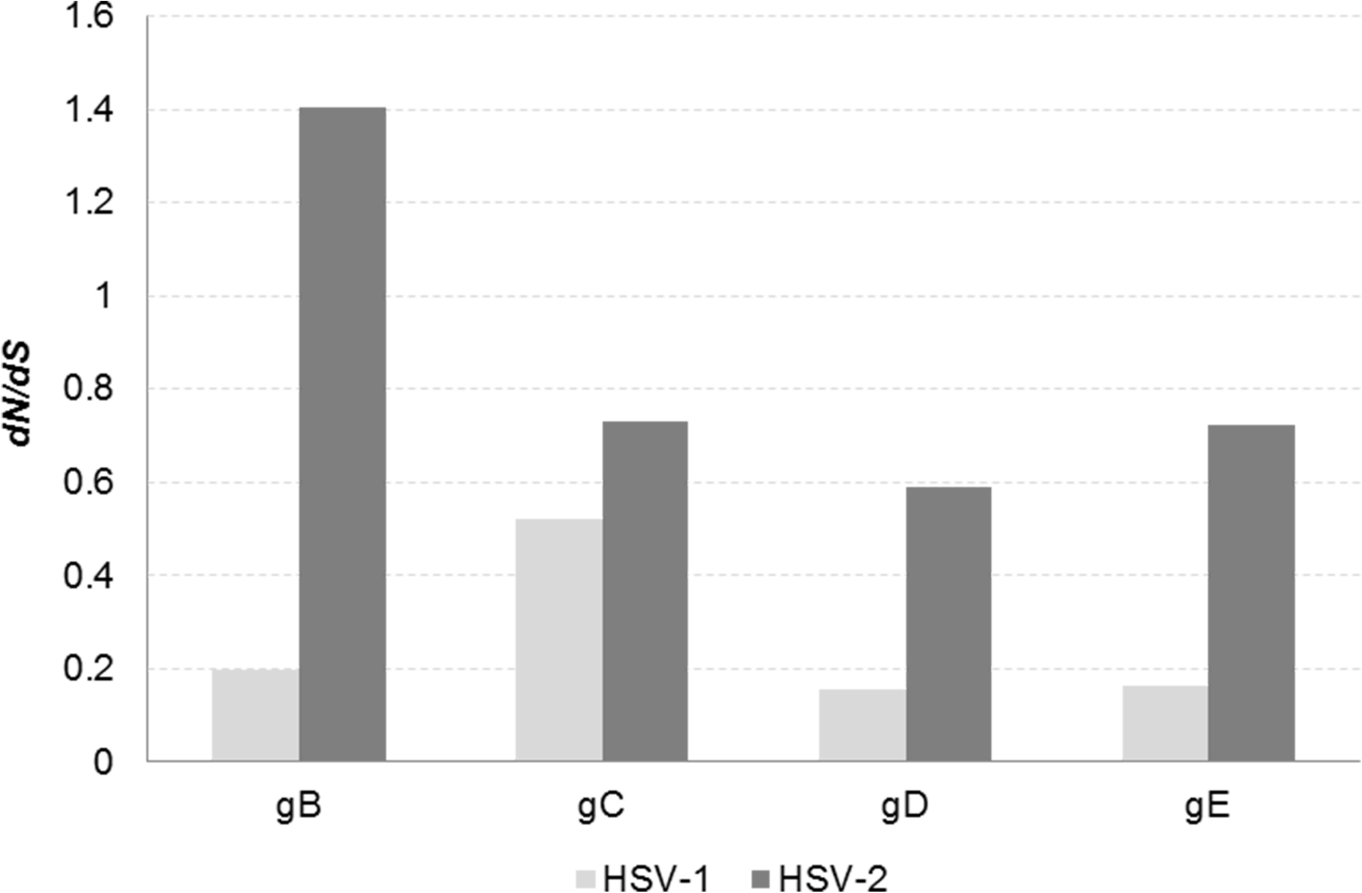
*d*N/*d*S ratios for HSV-1 and HSV-2 glycoprotein gene sequences. Mean ratio of non-synonymous to synonymous evolutionary substitutions (*d*N/*d*S) within the U_S_27 (gB), U_L_44 (gC), U_S_6 (gD) and U_S_8 (gE) genes is shown for all HSV-1 and -2 strains sampled. The *d*N/*d*S ratio for all HSV-2 gD sequences from gD-2 vaccine recipients was not significantly different than control subjects (0.61 versus 0.53, P = 0.554 by unpaired *t* test).

It was of interest to determine whether the vaccine may have placed immune selective pressure on infecting viruses such that the variant glycoprotein sequences most apt to evade pre-existing antibody to the gD ectodomain emerged. The *d*N/*d*S ratio measures the relative importance of selection as a driving force for amino acid changes in a coding region. We therefore determined this ratio for each of the glycoprotein sets. As shown in Fig 5 and S2 Table, most of the glycoproteins had *d*N/*d*S <1, indicating purifying selection pressure. The gD sequences of both HSV-1 and HSV-2 were highly constrained, as might be expected of a viral cell attachment protein. Higher *d*N/*d*S ratios were found for the HSV-2 glycoproteins, but only HSV-2 gB had *d*N/*d*S >1, suggesting positive selection. Even so, several methods of analysis failed to consistently identify positively selected residues (S3 Table).

## Discussion

The gD sequences of viral isolates derived from 83 women who became infected during the phase 3 Herpevac Trial were determined. Thirty-six of 44 HSV-2-infected subjects had gD sequences with an A353V substitution in the transmembrane domain compared with HSV-2 strain G from which the vaccine was derived. However, amino acid 353 was not contained in the truncated gD-2 vaccine so an immune response to the vaccine could not have exerted selective pressure. Similarly, 21 of 39 subjects’ HSV-1 isolates had H365R plus R369Q polymorphisms in the transmembrane and cytoplasmic regions not present in the vaccine. An E142D substitution in the ectodomain of several HSV-1 isolates and V169A in several HSV-2 isolates may be naturally occurring polymorphisms because they were also noted in patients’ gD sequences previously submitted to GenBank. Evidence that all of the above-mentioned substitutions could be naturally occurring polymorphisms include 1) the fact that the same nucleotide was substituted at these polymorphic sites in almost all instances; 2) most changes had also been found in one or more reference sequences; and 3) V169A and A353V were also noted in HSV-2 gD sequences from HIV positive or negative individuals [29]. Inspection of chromatograms did not reveal any overlapping peaks, suggesting that other sequence variants, if present, occurred at very low frequency. In addition, we observed complete conservation of nucleotide sequences between primary and recurrent isolates from the same subject, suggesting polymorphic residues were not a response to immune selective pressure post-infection. Therefore, evolution of gD is very constrained and gD sequence variation is not an explanation for the observed lack of vaccine efficacy against HSV-2 [29,59].

Different faces of the gD glycoprotein determine its critical functions in HSV infection (Fig 1A) [14]. One face interacts with the cellular receptor, and another interacts with gH/gL, causing a conformational change that renders gB fusion-competent [37]. The gD receptor HVEM [61] is found on lymphocytes [62], and the receptor nectin-1 [63] is a component of epithelial adherens junctions [64]. HSV-1 can also utilize *3-O*-sulfated heparin sulfate [65], and HSV-2 also utilizes nectin-2 [66]. Structural studies of gD bound to its receptor reveal displacement of the C terminus of the gD ectodomain, necessary for activating fusion via gH/gL and gB [15,67]. A V231W substitution in gD was found to mimic this displacement in the absence of receptor binding [68], and interestingly we observed one HSV-2 sequence with a V231I substitution. A37V and L47H are not residues directly involved in interaction with HVEM [69], and no residues in the N- or C-terminal extensions of gD known to interact with nectin-1 [70] are impacted by a substitution, as might be expected of viruses that had successfully infected subjects. Similarly, gD interactions with gH/gL [71] and gB [72] do not appear to be affected by any of the substitutions found in subjects’ isolates in the gD profusion domain. The A353V and A355M mutations lie within the gD transmembrane region and so are less likely to have affected gH/gL or gB interaction.

Point mutations that disrupt linear or discontinuous neutralizing antibody epitopes would permit escape from vaccine-elicited responses. The molecular interactions of one neutralizing antibody, E317, which binds a conformational epitope on gD have been solved by co-crystallization [36]. None of the amino acid changes in HSV-1 or HSV-2 gD sequences from Herpevac Trial subjects map to E317 contact residues. Linear epitopes on gD recognized by neutralizing antibody are represented by peptides 1–20, 10–29, 19–38, 262–281, and 280–316 [37,38]. A37V, which occurred in HSV-2 isolates of gD vaccine recipients who became infected, lies within linear epitope 1–20 (as numbered from the first amino acid after the signal sequence). The L47H mutation in one subject’s HSV-1 isolate lies within overlapping peptide 10–29, known to be recognized by patient sera [38]. Each of these substitutions could have contributed to immune evasion by preventing neutralizing antibody from binding and blocking the interaction of gD with its receptor [37]. Antibodies to this N-terminal region are found in the sera of HSV-1 and HSV-2-infected individuals [73,74], indicating this site is indeed immunogenic. However, the L47H and A37V mutations were unique among changes in single isolates and therefore could not have been a general cause of immune escape. It is possible that other point mutations or polymorphisms in some of the isolates cause conformational changes in gD which disrupt antibody-gD interaction. It will be of interest to determine the relative level of neutralizing antibody in these subjects’ sera to address the hypothesis that one or more of these mutations could have allowed the virus to escape neutralization.

Other potential explanations exist for why a gD-specific immune response to vaccine could not prevent infection despite overt similarity in amino acid sequence of the ectodomain: First, conformation of a protein can affect the binding capacity of antibody molecules whose epitopes are dependent on tertiary structure. It is possible that the polypeptide used for the vaccination adopts a different conformation than the same sequence when contained within the full-length protein. In addition, chemical composition of the adjuvant could potentially affect gD conformation. Second, polymorphisms may exist in a minority of reads that would not be visible as overlapping peaks in the Sanger sequence traces; however, any such minor variants most likely could not have contributed appreciably to capacity of these viruses to infect vaccinated individuals. Third, we focused on polymorphisms in the ectodomain of gD, but approximately half of HSV-1 isolates contained variant H365R plus R369Q residues in the cytoplasmic of the gD molecule. Conceivably polymorphic residues in this region could affect recruitment of gD to rafts, interactions with other viral proteins in the infected cell membrane, or vesicular transport in neurons. Lastly, although T cell responses did not correlate with protection in the Herpevac Trial (32), alteration of a CD4 or CD8 T cell epitope in gD could conceivably have allowed virus to evade a vaccine-induced T cell response to gD in certain subjects. No amino acid substitutions occurred within known CD8 T cell epitopes presented by HLA-A*0201 [75]. However, isolates from four HSV-2-infected, Herpevac Trial subjects contained a polymorphism (V169A) in a known HLA-DR-restricted, CD4 T cell epitope [76]. Three of the four isolates were from gD vaccine recipients. Whether this polymorphism could have partially reduced the CD4 T cell response in select subject(s) with an appropriate HLA-DR haplotype is not yet known.

A quantitative measure of vaccine-mediated selection of virus variants that could potentially evade the gD-specific immune response would be a finding of a higher *d*N/*d*S ratio for gD sequences from vaccine recipients compared with control-vaccinated subjects. Indeed, 8 out of the 10 individual HSV-2 gD sequences with *d*N/*d*S >1 came from gD-vaccinated subjects (P = 0.023). However, the *d*N/*d*S ratio for all HSV-2 gD sequences from gD-2 vaccine recipients was not significantly different than control subjects (0.61 versus 0.53, P = 0.554). In addition, the *d*N/*d*S ratios for gD sequences from the HSV-1 and HSV-2 isolates (S2 Table) align with previously published ratios for the same genes [27,28,77]. Overall, these results do not strongly support a specific capacity of the vaccine-induced anti-gD response to select natural sequence variants.

We identified A353V as a major discriminator between strains similar to HSV-2 strain G gD and those similar to SD90e. In a comparison of 36 geographically disparate HSV-2 gD sequences, amino acid 353 of HSV-2 gD had been identified as undergoing positive selection by the iFEL method [28], and as a polymorphic residue by a third group [29]. In our data set, residue 353 was not flagged as undergoing positive selection by any of the 5 methods tested (S3 Table). Interestingly, in the subject isolates we tested, either H365/R369 or R365/Q369 always occur together in the gD sequence, suggesting co-selection of this amino acid pair.

Definition of a reference strain is an important consideration in sequence comparisons. Our survey of 100 isolates from 83 subjects suggests that for HSV-1 gD, the sequence of laboratory strain KOS most closely resembled a significant proportion of the isolates. gD of McKrae represented a majority of the remaining isolates (14 at the amino acid level and 4 at the nucleotide level) due to the presence of the H365R/R369Q variant. Interestingly, nucleotide changes underlying these variant amino acids are also associated with a synonymous a>g substitution at position 963. Patterns of amino acid substitutions such as this variant cluster may be useful in epidemiological tracking, and also in assessing recombination frequency since the A4T substitution was negatively associated with the C-terminal cluster of substitutions. It has been argued that the South African strain SD90e should be used as the standard HSV-2 reference sequence [77]. Only 2 out of 44 subjects’ HSV-2 gD nucleotide sequences were identical to SD90e, and only 2 matched that of HG52 or strain G which was the source of the vaccine. However, SD90e contains nucleotides C733 and T1058 which characterize the majority of the 44 subjects’ gD sequences, and result in amino acid sequence identity with 27 of the 44 subjects. Greater identity with the South African strain than with the laboratory strain HG52 was also noted in HSV-2 gE and gB. Thus we chose SD90e as the reference sequence for HSV-2 gB, gC and gE.

Consideration of strain-dependent differences is also critical in vaccine design, as was demonstrated by the reduced capacity of *dl*5-29 to protect against South African strains compared to U.S. strains in a murine model [78]. For selection of a gD-based vaccine, our results suggest that the strain G ectodomain would adequately represent the ectodomain of most wild viruses since it was identical to 37 of 44 subjects’ gD ectodomains and to gD of SD90e.

Diversity among HSV-1 strains is described as greater than HSV-2 [79–81], and we found this to be true of the HSV-1 genes sampled here (Table 3). HSV-1 UL44 encoding gC was previously noted as among the most variable genes in the HSV-1 genome [27]. Consistent with this, we found significant variation among the subjects’ isolates, predominantly in the N-terminal half of the protein. Similarly, most HSV-1 gE sequences contained numerous amino acid substitutions relative to gE of KOS. Though our sample size was small, no consistent differences were readily apparent between sequences derived from infected gD-2 vaccine recipients and control recipients. In contrast, the HSV-2 gC and gE sequences we determined remained relatively uniform. The S123F substitution was previously observed in 4 of 5 HSV-2 gC genes sequenced [82], in a background of similarly low nucleotide and amino acid variation. The one exception was a highly polymorphic HSV-2 gC from a control recipient (Fig 2B). The higher affinity of HSV-2 gC for C3b binding than HSV-1 gC [83] may constrain its sequence variation if prevention of complement-mediated lysis is especially important to HSV-2 success. Whether subtle conformational alterations in HSV-1 gC and/or gE change the degree to which gD on the virion is shielded from antibody binding [25] remains to be determined, but our data thus far do not support the hypothesis that viruses infecting vaccine recipients were successful because of alterations in these glycoproteins.

HSV-2 gB is more variable than HSV-2 gD, as was previously found in another study of HIV-1/HSV-2 co-infected individuals [29]. In that study, HSV-2 gD amino acid sequences contained no or only 1 amino acid substitution (with 0 to 3 nucleotide changes), whereas gB sequences varied by an average of 2 to 7 amino acids per strain (and 4 to 10 nucleotides), a level of variation equivalent to what we observed (Fig 3B). Previous observation of R46Q, K57R, R327Q and Q393E substitutions in HSV-2 gB among one or more primary clinical isolates [82] implies that these may be naturally occurring polymorphisms. Even greater amino acid variation could be found in HSV-1 gB sequences of isolates from the Herpevac Trial, particularly in the N-terminus. The N-terminal variation in gB occurs in functional region IV of the molecule, whose flexibility has defied crystallization [84,85]. Intriguingly, compared with the genital isolates from the Herpevac Trial, the oral isolates we sequenced all contained numerous substitutions between amino acids 59 and 77, including 3 proline residues [86]. The substitutions in this N-terminal region and implications of the predicted conformational changes they cause include possible alteration of a continuous antibody epitope mapped within this region (T37A within epitope 31–43) [86,87] and possible subtle alterations in fusion activity [88]. The frequency of non-synonymous changes in HSV-1 gB suggests amino acid variation may help HSV maintain an advantage over its host in the face of an immune response, though whether that includes a vaccine-induced response to gD alone is still a matter of conjecture worthy of further exploration. Knowledge of inter-strain variations in gB and other viral proteins will permit refinement of vaccine antigens to generate a robust and broadly cross-reactive immune response.

Our sets of gD sequences from the Herpevac Trial dramatically increase the total number of gD sequences available for HSV-1 and HSV-2 primary isolates, particularly those from North America. HSV-2 gC sequences obtained in this study double the total number available for analysis, and the HSV-2 gE sequences represent the first from North American primary isolates. The fact that so many of the primary isolate glycoprotein sequences currently deposited in GenBank derive from other continents (Europe, Africa and Asia) may contribute to the different frequencies of polymorphic nucleotides in HSV-1 gD and gE when compared with the sequences in our study (Table 3) because we used reference sequence HSV-1 KOS (a U.S. strain). It is also possible, however, that the immune response to gD-2 vaccine may have subtly constrained the variation in wild-type HSV-1 strains able to successfully infect Herpevac Trial participants. Some Ts/Tv ratios were lower or higher than the expected range of 2 to 3 for coding regions of human genes [89], deviations previously observed for select HSV genes [27,49,90]. Because twice as many transversions as transitions are possible, a lower Ts/Tv ratio implies greater probability of transversional substitution even though high G+C content of HSV genes and hypermethylation of CpG dinucleotides would bias toward an elevated transition rate. Codon usage bias [91] and activities of DNA glycosylase and other mismatch repair enzymes may also influence the ratio.

In summary, gD sequence variation was highly constrained. Although numerous amino acid changes occurred in gC, gE, and gB relative to the reference sequences, especially for HSV-1, no consistent changes were identified that could be a correlate of successful infection. Comparison of ADCC activity or neutralizing antibody target(s) against viruses with glycoprotein variants, and in vaccinated infected subjects versus infected control recipients, may provide insights to guide further development of herpes simplex vaccines.

## Supporting information

S1 Table Genomes and accession numbers. (DOCX)

S2 Table Ratio of non-synonymous to synonymous evolutionary substitutions (*d*N/*d*S ratio) within the US27 (gB), UL44 (gC), US6 (gD) and US8 (gE) genes for all HSV-1 and -2 strains sampled. (DOCX)

S3 Table Amino acid positions under selection for HSV-1 and HSV-2 using five methods of analysis. (DOCX)
