# Supplementary material for: The herpevac trial for women: Sequence analysis of glycoproteins from viruses obtained from infected subjects
Source: PLoS One. 2017 Apr 27;12(4):e0176687. doi: 10.1371/journal.pone.0176687 (PMC5407825; doi:10.1371/journal.pone.0176687)
Supplement: S1 Table — (DOCX) [file pone.0176687.s001.docx]

| **S1 Table. Genomes and accession numbers.** | | | | | | | | | |
| --- | --- | --- | --- | --- | --- | --- | --- | --- | --- |
| **Strain** | **Virus** | **Anatomical site** | **Primary clinical isolate** | **Collection year** | **Geographic origin** | **GenBank accession number** | | | |
|  |  |  |  |  |  | **gB (U_L_27)** | **gC (U_L_44)** | **gD (U_S_6)** | **gE (U_S_8)** |
| KOS | HSV-1 | Lip | No | 1964 | United States | JQ673480 | JQ673480 | JQ673480 | JQ673480 |
| McKrae | HSV-1 | Eye | No | 1965 | United States | JX142173 | JX142173 | JX142173 | JX142173 |
| 17 | HSV-1 | na | No | 1972 | Scotland | FJ593289 | FJ593289 | FJ593289 | FJ593289 |
| F | HSV-1 | Lip | No | 1967 | United States | - | - | AF293614 | - |
| H129 | HSV-1 | Brain | Yes | 1977 | United States | GU734772 | GU734772 | GU734772 | GU734772 |
| Isolate 0116209 | HSV-1 | na | Yes | 2011 | India | KJ847330 | KJ847330 | KJ847330 | - |
| CR38 | HSV-1 | na | Yes | 1980 | China | HM585508 | HM585508 | HM585508 | HM585508 |
| E03 | HSV-1 | na | Yes | na | Kenya | HM585509 | HM585509 | HM585509 | HM585509 |
| E06 | HSV-1 | na | Yes | na | Kenya | HM585496 | HM585496 | HM585496 | HM585496 |
| E07 | HSV-1 | na | Yes | na | Kenya | HM585497 | HM585497 | HM585497 | HM585497 |
| E08 | HSV-1 | na | Yes | na | Kenya | HM585498 | HM585498 | HM585498 | HM585498 |
| E10 | HSV-1 | na | Yes | na | Kenya | HM585499 | HM585499 | HM585499 | HM585499 |
| E11 | HSV-1 | na | Yes | na | Kenya | HM585500 | HM585500 | HM585500 | HM585500 |
| E12 | HSV-1 | na | Yes | na | Kenya | HM585501 | HM585501 | HM585501 | HM585501 |
| E13 | HSV-1 | na | Yes | na | Kenya | HM585502 | HM585502 | HM585502 | HM585502 |
| E14 | HSV-1 | na | Yes | na | Kenya | HM585510 | HM585510 | HM585510 | HM585510 |
| E15 | HSV-1 | na | Yes | na | Kenya | HM585503 | HM585503 | HM585503 | HM585503 |
| E19 | HSV-1 | na | Yes | na | Kenya | HM585511 | HM585511 | HM585511 | HM585511 |
| E22 | HSV-1 | na | Yes | na | Kenya | HM585504 | HM585504 | HM585504 | HM585504 |
| E23 | HSV-1 | na | Yes | na | Kenya | HM585505 | HM585505 | HM585505 | HM585505 |
| E25 | HSV-1 | na | Yes | na | Kenya | HM585506 | HM585506 | HM585506 | HM585506 |
| E35 | HSV-1 | na | Yes | na | Kenya | HM585507 | HM585507 | HM585507 | HM585507 |
| R11 | HSV-1 | na | Yes | na | South Korea | HM585514 | HM585514 | HM585514 | HM585514 |
| R62 | HSV-1 | na | Yes | na | South Korea | HM585515 | HM585515 | HM585515 | HM585515 |
| S23 | HSV-1 | na | Yes | na | Japan | HM585512 | HM585512 | HM585512 | HM585512 |
| S25 | HSV-1 | na | Yes | na | Japan | HM585513 | HM585513 | HM585513 | HM585513 |
| O1 | HSV-1 | Oral | Yes | 2001 | Sweden | - | AJ421487 | - | - |
| O2 | HSV-1 | Oral | Yes | 2001 | Sweden | - | AJ421488 | - | - |
| O3 | HSV-1 | Oral | Yes | 2001 | Sweden | - | AJ421489 | - | - |
| 04/90132 | HSV-1 | Brain | Yes | 2001 | Sweden | - | AJ421490 | - | - |
| 05/90147 | HSV-1 | Brain | Yes | 2001 | Sweden | - | AJ421491 | - | - |
| 06/90237 | HSV-1 | Brain | Yes | 2001 | Sweden | - | AJ421492 | - | - |
| 07/90238 | HSV-1 | Brain | Yes | 2001 | Sweden | - | AJ421493 | - | - |
| 08/90395 | HSV-1 | Brain | Yes | 2001 | Sweden | - | AJ421494 | - | - |
| 09/90579 | HSV-1 | Brain | Yes | 2001 | Sweden | - | AJ421495 | - | - |
| 010/90602 | HSV-1 | Brain | Yes | 2001 | Sweden | - | AJ421496 | - | - |
| 011/94783 | HSV-1 | Brain | Yes | 2001 | Sweden | - | AJ421497 | - | - |
| G1 | HSV-1 | Genital | Yes | 2001 | Sweden | - | AJ421498 | - | - |
| G2 | HSV-1 | Genital | Yes | 2001 | Sweden | - | AJ421499 | - | - |
| G3 | HSV-1 | Genital | Yes | 2001 | Sweden | - | AJ421500 | - | - |
| 274 | HSV-1 | Brain | Yes | 2001 | Sweden | - | AJ421501 | - | - |
| 1666 | HSV-1 | Brain | Yes | 2001 | Sweden | - | AJ421502 | - | - |
| 3355 | HSV-1 | Brain | Yes | 2001 | Sweden | - | AJ421503 | - | - |
| 7682 | HSV-1 | Brain | Yes | 2001 | Sweden | - | AJ421504 | - | - |
| E4 | HSV-1 | Brain | Yes | 2001 | Sweden | - | AJ421505 | - | - |
| 2762 | HSV-1 | Brain | Yes | 2001 | Sweden | - | AJ421506 | - | - |
| 25 | HSV-1 | Brain | Yes | 2001 | Sweden | - | AJ421507 | - | - |
| E5 | HSV-1 | Brain | Yes | 2001 | Sweden | - | AJ421508 | - | - |
| BAN | HSV-1 | Brain | Yes | 2001 | Sweden | - | AJ421509 | - | - |
| Isolate_VRF_54 | HSV-1 | Genital | Yes | 2012 | India | - | JN712694 | - | - |
| Isolate_VRF_320 | HSV-1 | Genital | Yes | 2012 | India | - | JN712695 | - | - |
| Isolate_VRF_416 | HSV-1 | Eye | Yes | 2012 | India | - | JN712697 | - | - |
| Isolate_VRF_342 | HSV-1 | Genital | Yes | 2012 | India | - | JN712696 | - | - |
| Isolate_VRF_666 | HSV-1 | Skin | Yes | 2012 | India | - | JN712698 | - | - |
| Isolate_VRF_689 | HSV-1 | Throat | Yes | 2012 | India | - | JN712699 | - | - |
| Isolate_VRF_922 | HSV-1 | Eye | Yes | 2012 | India | - | JN712700 | - | - |
| Isolate_VRF_1105 | HSV-1 | Eye | Yes | 2012 | India | - | JN712701 | - | - |
| Isolate_VRF_1217 | HSV-1 | Throat | Yes | 2012 | India | - | JN712702 | - | - |
| Isolate_VRF_1362 | HSV-1 | Eye | Yes | 2012 | India | - | JN712703 | - | - |
| Isolate_VRF_1464 | HSV-1 | Eye | Yes | 2012 | India | - | JN712704 | - | - |
| Isolate_VRF_1486 | HSV-1 | Ulcer | Yes | 2012 | India | - | JN712705 | - | - |
| Isolate_VRF_2857 | HSV-1 | Eye | Yes | 2012 | India | - | JN712706 | - | - |
| Isolate_VRF_2889 | HSV-1 | Lip | Yes | 2012 | India | - | JN712707 | - | - |
| Isolate_VRF_3341 | HSV-1 | Eye | Yes | 2012 | India | - | JN712708 | - | - |
| Isolate_VRF_3837 | HSV-1 | Eye | Yes | 2012 | India | - | JN712709 | - | - |
| Isolate_VRF_GH01 | HSV-1 | Lip | Yes | 2012 | India | - | JN712710 | - | - |
| Isolate_VRF_GH03 | HSV-1 | Ulcer | Yes | 2012 | India | - | JN712711 | - | - |
| Isolate_VRF_AEH02 | HSV-1 | Eye | Yes | 2012 | India | - | JN712712 | - | - |
| Isolate_VRF_AEH01 | HSV-1 | Eye | Yes | 2012 | India | - | JN712713 | - | - |
| Isolate_VRF_AEH12 | HSV-1 | Eye | Yes | 2012 | India | - | JN712714 | - | - |
| Isolate_VRF_SN01 | HSV-1 | Eye | Yes | 2012 | India | - | JN712715 | - | - |
| Isolate_VRF_SN02 | HSV-1 | Eye | Yes | 2012 | India | - | JN712716 | - | - |
| Isolate_VRF_2307 | HSV-1 | Eye | Yes | 2012 | India | - | JN712717 | - | - |
| Isolate_VRF_4184 | HSV-1 | Vitreous Aspirate | Yes | 2012 | India | - | JN712718 | - | - |
| Isolate_E5 | HSV-1 | na | Yes | 1998-2012 | Brazil | - | KM279072 | - | KM279046 |
| Isolate_E23 | HSV-1 | na | Yes | 1998-2012 | Brazil | - | KM279091 | - | KM279048 |
| Isolate_KHS2 | HSV-1 | na | Yes | na | Korea | - | - | AF487902 | - |
| Isolate_KHS1 | HSV-1 | na | Yes | na | Korea | - | - | AF487901 | - |
| Isolate_E4 | HSV-1 | na | Yes | 1998-2012 | Brazil | - | - | - | KM279050 |
| Isolate_E10 | HSV-1 | na | Yes | 1998-2012 | Brazil | - | - | - | KM279049 |
| Isolate_I18 | HSV-1 | na | Yes | 1998-2012 | Brazil | - | - | - | KM279047 |
| Isolate_E67 | HSV-1 | na | Yes | 1998-2012 | Brazil | - | - | - | KM279045 |
| Sample1 | HSV-1 | Genital | Yes | 2006 | United States | - | - | **KY223523** | - |
| Sample2 | HSV-1 | Genital | Yes | 2003 | United States | **KY274362** | **KY274370** | **KY223524** | **KY274378** |
| Sample3 | HSV-1 | Genital | Yes | 2007 | United States | **KY274363** | **KY274371** | **KY223525** | **KY274379** |
| Sample4 | HSV-1 | Genital | Yes | 2004 | United States | - | - | **KY223526** | - |
| Sample5 | HSV-1 | Genital | Yes | 2007 | United States | **KY274364** | **KY274372** | **KY223527** | **KY274380** |
| Sample7 | HSV-1 | Oral | Yes | 2006 | United States | **KY274365** | **KY274373** | **KY223528** | **KY274381** |
| Sample9 | HSV-1 | Genital | Yes | 2005 | United States | - | - | **KY223529** | - |
| Sample11 | HSV-1 | Oral | Yes | 2008 | United States | **KY274366** | **KY274374** | **KY223530** | **KY274382** |
| Sample12 | HSV-1 | Genital | Yes | 2007 | United States | - | - | **KY223531** | - |
| Sample20 | HSV-1 | Oral | Yes | 2005 | United States | **KY274367** | **KY274375** | **KY223532** | **KY274383** |
| Isolate1-1 | HSV-1 | Genital | Yes | 2005 | United States | - | - | **KY223533** | - |
| Isolate1-3 | HSV-1 | Genital | Yes | 2008 | United States | - | - | **KY223534** | - |
| Isolate1-4 | HSV-1 | Oral | Yes | 2003 | United States | - | - | **KY223535** | - |
| Isolate1-6 | HSV-1 | Oral | Yes | 2004 | United States | - | - | **KY223536** | - |
| Isolate1-7 | HSV-1 | Genital | Yes | 2006 | United States | - | - | **KY223537** | - |
| Isolate1-8 | HSV-1 | Genital | Yes | 2008 | United States | - | - | **KY223538** | - |
| Isolate1-9 | HSV-1 | Genital | Yes | 2005 | United States | - | - | **KY223539** | - |
| Isolate1-11 | HSV-1 | Genital | Yes | 2004 | United States | - | - | **KY223540** | - |
| Isolate1-12 | HSV-1 | Oral | Yes | 2006 | United States | - | - | **KY223541** | - |
| Isolate1-13 | HSV-1 | Genital | Yes | 2005 | United States | - | - | **KY223542** | - |
| Isolate1-14 | HSV-1 | Oral | Yes | 2007 | United States | - | - | **KY223543** | - |
| Isolate1-15 | HSV-1 | Oral | Yes | 2007 | United States | **KY274368** | **KY274376** | **KY223544** | **KY274384** |
| Isolate1-16 | HSV-1 | Genital | Yes | 2006 | United States | **KY274369** | **KY274377** | - | **KY274385** |
| Isolate1-17 | HSV-1 | Genital | Yes | 2007 | United States | - | - | **KY223545** | - |
| Isolate1-18 | HSV-1 | Oral | Yes | 2005 | United States | - | - | **KY223546** | - |
| Isolate1-20 | HSV-1 | Genital | Yes | 2007 | United States | - | - | **KY223547** | - |
| Isolate1-22 | HSV-1 | Genital | Yes | 2007 | United States | - | - | **KY223548** | - |
| Isolate1-24 | HSV-1 | Genital | Yes | 2005 | United States | - | - | **KY223549** | - |
| Isolate1-25 | HSV-1 | Oral | Yes | 2006 | United States | - | - | **KY223550** | - |
| Isolate1-26 | HSV-1 | Genital | Yes | 2007 | United States | - | - | **KY223551** | - |
| Isolate1-27 | HSV-1 | Genital | Yes | 2005 | United States | - | - | **KY223552** | - |
| Isolate1-28 | HSV-1 | Genital | Yes | 2005 | United States | - | - | **KY223553** | - |
| Isolate1-29 | HSV-1 | Genital | Yes | 2005 | United States | - | - | **KY223554** | - |
| Isolate1-30 | HSV-1 | Rectal | Yes | 2006 | United States | - | - | **KY223555** | - |
| Isolate1-31 | HSV-1 | Genital | Yes | 2006 | United States | - | - | **KY223556** | - |
| Isolate1-32 | HSV-1 | Genital | Yes | 2006 | United States | - | - | **KY223557** | - |
| Isolate1-34 | HSV-1 | Genital | Yes | 2007 | United States | - | - | **KY223558** | - |
| Isolate1-35 | HSV-1 | Rectal | Yes | 2008 | United States | - | - | **KY223559** | - |
| Isolate1-36 | HSV-1 | Genital | Yes | 2008 | United States | - | - | **KY223560** | - |
| Isolate1-37 | HSV-1 | Genital | Yes | 2007 | United States | - | - | **KY223561** | - |
| HG52 | HSV-2 | Anal | No | Prior to 1971 | Scotland | JN561323 | JN561323 | JN561323 | JN561323 |
| SD90e | HSV-2 | Genital | Yes | 1994 | South Africa | KF781518 | KF781518 | KF781518 | KF781518 |
| 333 | HSV-2 | Genital | No | 1971 | United States | M24771 | X01996 | EU018091 | EU018094 |
| G | HSV-2 | Genital | No | 1967 | United States | - | - | KY933650 | - |
| 186 | HSV-2 | Genital | No | 1968 | na | - | - | JX112656 | - |
| Isolate_99_62039 | HSV-2 | Genital | Yes | 1999 | Germany | HM011366 | - | - | - |
| Isolate_99_59340 | HSV-2 | Genital | Yes | 1999 | Germany | HM011365 | - | - | - |
| Isolate_99_30884 | HSV-2 | Genital | Yes | 1999 | Germany | HM011364 | - | - | - |
| Isolate_99_30304 | HSV-2 | Genital | Yes | 1999 | Germany | HM011363 | - | - | - |
| Isolate_99_30262 | HSV-2 | Genital | Yes | 1999 | Germany | HM011362 | - | - | - |
| Isolate_99_12518 | HSV-2 | Genital | Yes | 1999 | Germany | HM011361 | - | - | - |
| Isolate_98_60866 | HSV-2 | Genital | Yes | 1998 | Germany | HM011360 | - | - | - |
| Isolate_98_17422 | HSV-2 | Genital | Yes | 1998 | Germany | HM011359 | - | - | - |
| Isolate_98_15754 | HSV-2 | Genital | Yes | 1998 | Germany | HM011358 | - | - | - |
| Isolate_98_15713 | HSV-2 | Genital | Yes | 1998 | Germany | HM011357 | - | - | - |
| Isolate_98_15085 | HSV-2 | Genital | Yes | 1998 | Germany | HM011356 | - | - | - |
| Isolate_98_12141 | HSV-2 | Genital | Yes | 1998 | Germany | HM011355 | - | - | - |
| Isolate_98_04792 | HSV-2 | Genital | Yes | 1998 | Germany | HM011354 | - | - | - |
| Isolate_98_04616 | HSV-2 | Genital | Yes | 1998 | Germany | HM011353 | - | - | - |
| Isolate_97_51631 | HSV-2 | Genital | Yes | 1997 | Germany | HM011351 | - | - | - |
| Isolate_97_43891 | HSV-2 | Genital | Yes | 1997 | Germany | HM011350 | - | - | - |
| Isolate_97_43688 | HSV-2 | Genital | Yes | 1997 | Germany | HM011349 | - | - | - |
| Isolate_97_42053 | HSV-2 | Genital | Yes | 1997 | Germany | HM011348 | - | - | - |
| Isolate_97_40665 | HSV-2 | Genital | Yes | 1997 | Germany | HM011347 | - | - | - |
| Isolate_97_40592 | HSV-2 | Genital | Yes | 1997 | Germany | HM011346 | - | - | - |
| Isolate_97_31214 | HSV-2 | Genital | Yes | 1997 | Germany | HM011345 | - | - | - |
| Isolate_08_16182 | HSV-2 | Genital | Yes | 2008 | Germany | HM011344 | - | - | - |
| Isolate_08_01808 | HSV-2 | Genital | Yes | 2008 | Germany | HM011343 | - | - | - |
| Isolate_08_09069 | HSV-2 | Genital | Yes | 2008 | Germany | HM011342 | - | - | - |
| Isolate_07_39034 | HSV-2 | Genital | Yes | 2008 | Germany | HM011341 | - | - | - |
| Isolate_07_26252 | HSV-2 | Genital | Yes | 2007 | Germany | HM011340 | - | - | - |
| Isolate_06_49144 | HSV-2 | Genital | Yes | 2006 | Germany | HM011339 | - | - | - |
| Isolate_06_25747 | HSV-2 | Genital | Yes | 2006 | Germany | HM011338 | - | - | - |
| Isolate_06_17037 | HSV-2 | Genital | Yes | 2006 | Germany | HM011337 | - | - | - |
| Isolate_06_14257 | HSV-2 | Genital | Yes | 2006 | Germany | HM011336 | - | - | - |
| Isolate_06_10066 | HSV-2 | Genital | Yes | 2006 | Germany | HM011335 | - | - | - |
| Isolate_05_53004 | HSV-2 | Genital | Yes | 2005 | Germany | HM011334 | - | - | - |
| Isolate_05_49495 | HSV-2 | Genital | Yes | 2005 | Germany | HM011333 | - | - | - |
| Isolate_05_38237 | HSV-2 | Genital | Yes | 2005 | Germany | HM011332 | - | - | - |
| Isolate_05_26435 | HSV-2 | Genital | Yes | 2005 | Germany | HM011331 | - | - | - |
| Isolate_05_26198 | HSV-2 | Genital | Yes | 2005 | Germany | HM011330 | - | - | - |
| Isolate_05_23936 | HSV-2 | Genital | Yes | 2005 | Germany | HM011329 | - | - | - |
| Isolate_05_06075 | HSV-2 | Genital | Yes | 2005 | Germany | HM011328 | - | - | - |
| Isolate_04_33071 | HSV-2 | Genital | Yes | 2004 | Germany | HM011327 | - | - | - |
| Isolate_04_11588 | HSV-2 | Genital | Yes | 2004 | Germany | HM011326 | - | - | - |
| Isolate_04_11851 | HSV-2 | Genital | Yes | 2004 | Germany | HM011325 | - | - | - |
| Isolate_04_10616 | HSV-2 | Genital | Yes | 2004 | Germany | HM011324 | - | - | - |
| Isolate_04_04007 | HSV-2 | Genital | Yes | 2004 | Germany | HM011323 | - | - | - |
| Isolate_04_01012 | HSV-2 | Genital | Yes | 2004 | Germany | HM011322 | - | - | - |
| Isolate_03_43378 | HSV-2 | Genital | Yes | 2003 | Germany | HM011321 | - | - | - |
| Isolate_03_39884 | HSV-2 | Genital | Yes | 2003 | Germany | HM011320 | - | - | - |
| Isolate_03_24861 | HSV-2 | Genital | Yes | 2003 | Germany | HM011319 | - | - | - |
| Isolate_03_21213 | HSV-2 | Genital | Yes | 2003 | Germany | HM011318 | - | - | - |
| Isolate_03_06516 | HSV-2 | Genital | Yes | 2003 | Germany | HM011317 | - | - | - |
| Isolate_03_06279 | HSV-2 | Genital | Yes | 2003 | Germany | HM011316 | - | - | - |
| Isolate_03_01982 | HSV-2 | Genital | Yes | 2003 | Germany | HM011315 | - | - | - |
| Isolate_02_27068 | HSV-2 | Genital | Yes | 2002 | Germany | HM011314 | - | - | - |
| Isolate_02_22326 | HSV-2 | Genital | Yes | 2002 | Germany | HM011313 | - | - | - |
| Isolate_02_17104 | HSV-2 | Genital | Yes | 2002 | Germany | HM011312 | - | - | - |
| Isolate_02_13690 | HSV-2 | Genital | Yes | 2002 | Germany | HM011311 | - | - | - |
| Isolate_02_13687 | HSV-2 | Genital | Yes | 2002 | Germany | HM011310 | - | - | - |
| Isolate_02_10179 | HSV-2 | Genital | Yes | 2002 | Germany | HM011309 | - | - | - |
| Isolate_01_49166 | HSV-2 | Genital | Yes | 2001 | Germany | HM011308 | - | - | - |
| Isolate_01_41463 | HSV-2 | Genital | Yes | 2001 | Germany | HM011307 | - | - | - |
| Isolate_00_29288 | HSV-2 | Genital | Yes | 2001 | Germany | HM011306 | - | - | - |
| Isolate_00_10414 | HSV-2 | Genital | Yes | 2000 | Germany | HM011305 | - | - | - |
| Isolate_00_10045 | HSV-2 | Genital | Yes | 2000 | Germany | HM011304 | - | - | - |
| Isolate_00_01097 | HSV-2 | Genital | Yes | 2000 | Germany | HM011303 | - | - | - |
| BBKC | HSV-2 | CSF | Yes | 1998 | United States | AF021340 | - | AF021342 | - |
| WTW1A | HSV-2 | Genital | Yes | 1998 | United States | U12175 | U12179 | U12183 | - |
| MMA | HSV-2 | Genital | Yes | 1998 | United States | U12174 | U12178 | U12182 | - |
| JDZ3 | HSV-2 | Throat | Yes | 1998 | United States | U12173 | U12177 | U12181 | - |
| CAM4B | HSV-2 | Perianal | Yes | 1998 | United States | U12172 | U12176 | U12180 | - |
| Isolate_ HSV-2v_pat12 | HSV-2 | Genital | Yes | 2006-2013 | Ivory Coast | KF588417 | - | KF588429 | - |
| Isolate_ HSV-2v_pat11 | HSV-2 | Buttock | Yes | 2006-2013 | Ivory Coast | KF588416 | - | KF588428 | - |
| Isolate_ HSV-2v_pat10 | HSV-2 | Genital | Yes | 2006-2013 | Congo | KF588415 | - | KF588427 | - |
| Isolate_ HSV-2v_pat9 | HSV-2 | Genital | Yes | 2006-2013 | Ivory Coast | KF588414 | - | KF588426 | - |
| Isolate_ HSV-2v_pat8 | HSV-2 | Genital | Yes | 2006-2013 | Mali | KF588413 | - | KF588425 | - |
| Isolate_ HSV-2v_pat7 | HSV-2 | Genital | Yes | 2006-2013 | Mali | KF588412 | - | KF588424 | - |
| Isolate_ HSV-2v_pat6 | HSV-2 | Buttock | Yes | 2006-2013 | Guinea | KF588411 | - | KF588423 | - |
| Isolate_ HSV-2v_pat5 | HSV-2 | Buttock | Yes | 2006-2013 | Africa (unspecified) | KF588410 | - | KF588422 | - |
| Isolate_ HSV-2v_pat4 | HSV-2 | Genital | Yes | 2006-2013 | Ivory Coast | KF588409 | - | KF588421 | - |
| Isolate_ HSV-2v_pat3 | HSV-2 | Anus | Yes | 2006-2013 | Nigeria | KF588408 | - | KF588420 | - |
| Isolate_ HSV-2v_pat2 | HSV-2 | Buttock | Yes | 2006-2013 | Niger | KF588407 | - | KF588419 | - |
| Isolate_ HSV-2v_pat1 | HSV-2 | Genital | Yes | 2006-2013 | Guinea | KF588406 | - | KF588418 | - |
| Isolate_ HSV-2v_pat14 | HSV-2 | Genital | Yes | 2006-2013 | Africa (unspecified) | KM068889 | - | KM068891 | - |
| Isolate_ HSV-2v_pat13 | HSV-2 | Buttock | Yes | 2006-2013 | Congo | KM068888 | - | KM068890 | - |
| Isolate_Pt26 | HSV-2 | Genital | Yes | 2007-2010 | France | JQ956350 | - | JQ956374 | - |
| Isolate_Pt25 | HSV-2 | Genital | Yes | 2007-2010 | France | JQ956349 | - | JQ956373 | - |
| Isolate_Pt24 | HSV-2 | Genital | Yes | 2007-2010 | France | JQ956348 | - | JQ956372 | - |
| Isolate_Pt23 | HSV-2 | Genital | Yes | 2007-2010 | France | JQ956347 | - | JQ956371 | - |
| Isolate_Pt22 | HSV-2 | Genital | Yes | 2007-2010 | France | JQ956346 | - | JQ956370 | - |
| Isolate_Pt21 | HSV-2 | Genital | Yes | 2007-2010 | France | JQ956345 | - | JQ956369 | - |
| Isolate_Pt20 | HSV-2 | Bronchi | Yes | 2007-2010 | France | JQ956344 | - | JQ956368 | - |
| Isolate_Pt19 | HSV-2 | Genital | Yes | 2007-2010 | France | JQ956343 | - | JQ956367 | - |
| Isolate_Pt18 | HSV-2 | Genital | Yes | 2007-2010 | France | JQ956342 | - | JQ956366 | - |
| Isolate_Pt16 | HSV-2 | Genital | Yes | 2007-2010 | France | JQ956341 | - | JQ956365 | - |
| Isolate_Pt15 | HSV-2 | Genital | Yes | 2007-2010 | France | JQ956340 | - | JQ956364 | - |
| Isolate_Pt13 | HSV-2 | Genital | Yes | 2007-2010 | France | JQ956338 | - | JQ956362 | - |
| Isolate_Pt14 | HSV-2 | Genital | Yes | 2007-2010 | France | JQ956339 | - | JQ956363 | - |
| Isolate_Pt12 | HSV-2 | Genital | Yes | 2007-2010 | France | JQ956337 | - | JQ956361 | - |
| Isolate_Pt11 | HSV-2 | Genital | Yes | 2007-2010 | France | JQ956336 | - | JQ956360 | -- |
| Isolate_Pt10 | HSV-2 | Genital | Yes | 2007-2010 | France | JQ956335 | - | JQ956359 | - |
| Isolate_Pt09 | HSV-2 | Genital | Yes | 2007-2010 | France | JQ956334 | - | JQ956358 | - |
| Isolate_Pt08 | HSV-2 | Genital | Yes | 2007-2010 | France | JQ956333 | - | JQ956357 | - |
| Isolate_Pt07 | HSV-2 | Genital | Yes | 2007-2010 | France | JQ956332 | - | JQ956356 | - |
| Isolate_Pt06 | HSV-2 | Genital | Yes | 2007-2010 | France | JQ956331 | - | JQ956355 | - |
| Isolate_Pt05 | HSV-2 | Genital | Yes | 2007-2010 | France | JQ956330 | - | JQ956354 | - |
| Isolate_Pt04 | HSV-2 | Genital | Yes | 2007-2010 | France | JQ956329 | - | JQ956353 | - |
| Isolate_Pt02 | HSV-2 | Genital | Yes | 2007-2010 | France | JQ956328 | - | JQ956352 | - |
| Isolate_Pt01 | HSV-2 | Genital | Yes | 2007-2010 | France | JQ956349 | - | - | - |
| VRC11098 | HSV-2 | Genital | Yes | 1988 | United States | - | - | EU029158 | - |
| 16293 | HSV-2 | Genital | Yes | 1988 | United States | - | - | AY779754 | - |
| 11449 | HSV-2 | Genital | Yes | 1988 | United States | - | - | AY779753 | - |
| 7566 | HSV-2 | Genital | Yes | 1988 | United States | - | - | AY779752 | - |
| 2899 | HSV-2 | Genital | Yes | 1988 | United States | - | - | AY779751 | - |
| 2589 | HSV-2 | Genital | Yes | 1988 | United States | - | - | AY779750 | - |
| Isolate_S_93_4530 | HSV-2 | Genital | Yes | na | Sweden | - | - | - | EU106468 |
| Isolate_S_95_580 | HSV-2 | Genital | Yes | na | Sweden | - | - | - | EU106467 |
| Isolate_S_97_1643 | HSV-2 | Genital | Yes | na | Sweden | - | - | - | EU106466 |
| Isolate_T_NN1 | HSV-2 | Genital | Yes | na | Tanzania | - | - | - | EU106458 |
| Isolate_T_64_3300 | HSV-2 | Genital | Yes | na | Tanzania | - | - | - | EU106457 |
| Isolate_T_70_3486 | HSV-2 | Genital | Yes | na | Tanzania | - | - | - | EU106456 |
| Isolate_T_56_3127 | HSV-2 | Genital | Yes | na | Tanzania | - | - | - | EU106455 |
| Isolate_T_53_1390 | HSV-2 | Genital | Yes | na | Tanzania | - | - | - | EU106454 |
| Isolate_T_50_3010 | HSV-2 | Genital | Yes | na | Tanzania | - | - | - | EU106453 |
| Isolate_T_47_2929 | HSV-2 | Genital | Yes | na | Tanzania | - | - | - | EU106452 |
| Isolate_T_44_2869 | HSV-2 | Genital | Yes | na | Tanzania | - | - | - | EU106451 |
| Isolate_T_43_742 | HSV-2 | Genital | Yes | na | Tanzania | - | - | - | EU106450 |
| Isolate_T_39_2801 | HSV-2 | Genital | Yes | na | Tanzania | - | - | - | EU106449 |
| Isolate_T_35_2769 | HSV-2 | Genital | Yes | na | Tanzania | - | - | - | EU106448 |
| Isolate_T_28_2595 | HSV-2 | Genital | Yes | na | Tanzania | - | - | - | EU106447 |
| Isolate_T_26_2794 | HSV-2 | Genital | Yes | na | Tanzania | - | - | - | EU106446 |
| Isolate_T_2346_14 | HSV-2 | Genital | Yes | na | Tanzania | - | - | - | EU106445 |
| Isolate_T_2316_10 | HSV-2 | Genital | Yes | na | Tanzania | - | - | - | EU106444 |
| Isolate_T_2228_01 | HSV-2 | Genital | Yes | na | Tanzania | - | - | - | EU106443 |
| Isolate_T_2211_12 | HSV-2 | Genital | Yes | na | Tanzania | - | - | - | EU106442 |
| Isolate_T_3041 | HSV-2 | Genital | Yes | na | Tanzania | - | - | - | EU106441 |
| Isolate_T_1855 | HSV-2 | Genital | Yes | na | Tanzania | - | - | - | EU106440 |
| Isolate_T_2032 | HSV-2 | Genital | Yes | na | Tanzania | - | - | - | EU106439 |
| Isolate_T_2737 | HSV-2 | Genital | Yes | na | Tanzania | - | - | - | EU106438 |
| Isolate_T_2357 | HSV-2 | Genital | Yes | na | Tanzania | - | - | - | EU106437 |
| Isolate_T_2557 | HSV-2 | Genital | Yes | na | Tanzania | - | - | - | EU106436 |
| Isolate_T_4587 | HSV-2 | Genital | Yes | na | Tanzania | - | - | - | EU106435 |
| Isolate_T_3111 | HSV-2 | Genital | Yes | na | Tanzania | - | - | - | EU106434 |
| Isolate_T_3034 | HSV-2 | Genital | Yes | na | Tanzania | - | - | - | EU106433 |
| Isolate_T_2302 | HSV-2 | Genital | Yes | na | Tanzania | - | - | - | EU106432 |
| Isolate_N_10 | HSV-2 | Genital | Yes | na | Norway | - | - | - | EU106431 |
| Isolate_N_9 | HSV-2 | Genital | Yes | na | Norway | - | - | - | EU106430 |
| Isolate_N_8 | HSV-2 | Genital | Yes | na | Norway | - | - | - | EU106429 |
| Isolate_N_7 | HSV-2 | Genital | Yes | na | Norway | - | - | - | EU106428 |
| Isolate_N_6 | HSV-2 | Genital | Yes | na | Norway | - | - | - | EU106427 |
| Isolate_N_5 | HSV-2 | Genital | Yes | na | Norway | - | - | - | EU106426 |
| Isolate_N_4 | HSV-2 | Genital | Yes | na | Norway | - | - | - | EU106425 |
| Isolate_N_3 | HSV-2 | Genital | Yes | na | Norway | - | - | - | EU106424 |
| Isolate_N_2 | HSV-2 | Genital | Yes | na | Norway | - | - | - | EU106423 |
| Isolate_N_1 | HSV-2 | Genital | Yes | na | Norway | - | - | - | EU106422 |
| Sample6 | HSV-2 | Genital | Yes | 2006 | United States | **KY274386** | **KY274392** | **KY223598** | **KY274398** |
| Sample8 | HSV-2 | Genital | Yes | 2004 | United States | **KY274387** | **KY274393** | **KY223599** | **KY274399** |
| Sample10 | HSV-2 | Genital | Yes | 2005 | United States | - | - | **KY223600** | - |
| Sample13 | HSV-2 | Genital | Yes | 2005 | United States | - | - | **KY223601** | - |
| Sample15 | HSV-2 | Genital | Yes | 2004 | United States | **KY274388** | **KY274394** | **KY223602** | **KY274400** |
| Sample16 | HSV-2 | Genital | Yes | 2007 | United States | **KY274389** | **KY274395** | **KY223603** | **KY274401** |
| Sample17 | HSV-2 | Genital | Yes | 2007 | United States | **KY274390** | **KY274396** | **KY223604** | **KY274402** |
| Sample18 | HSV-2 | Genital | Yes | 2006 | United States | **KY274391** | **KY274397** | **KY223605** | **KY274403** |
| Isolate2-1 | HSV-2 | Genital | Yes | 2004 | United States | - | - | **KY223562** | - |
| Isolate2-2 | HSV-2 | Genital | Yes | 2004 | United States | - | - | **KY223563** | - |
| Isolate2-3 | HSV-2 | Genital | Yes | 2004 | United States | - | - | **KY223564** | - |
| Isolate2-4 | HSV-2 | Genital | Yes | 2005 | United States | - | - | **KY223565** | - |
| Isolate2-6 | HSV-2 | Genital | Yes | 2006 | United States | - | - | **KY223566** | - |
| Isolate2-8 | HSV-2 | Genital | Yes | 2007 | United States | - | - | **KY223567** | - |
| Isolate2-11 | HSV-2 | Genital | Yes | 2003 | United States | - | - | **KY223568** | - |
| Isolate2-14 | HSV-2 | Genital | Yes | 2003 | United States | - | - | **KY223569** | - |
| Isolate2-15 | HSV-2 | Genital | Yes | 2007 | United States | - | - | **KY223570** | - |
| Isolate2-16 | HSV-2 | Genital | Yes | 2008 | United States | - | - | **KY223571** | - |
| Isolate2-18 | HSV-2 | Genital | Yes | 2004 | United States | - | - | **KY223572** | - |
| Isolate2-20 | HSV-2 | Genital | Yes | 2005 | United States | - | - | **KY223573** | - |
| Isolate2-21 | HSV-2 | Genital | No | 2004 | United States | - | - | **KY223574** | - |
| Isolate2-23 | HSV-2 | Genital | Yes | 2008 | United States | - | - | **KY223575** | - |
| Isolate2-24 | HSV-2 | Genital | No | 2004 | United States | - | - | **KY223576** | - |
| Isolate2-25 | HSV-2 | Genital | Yes | 2005 | United States | - | - | **KY223577** | - |
| Isolate2-26 | HSV-2 | Genital | Yes | 2005 | United States | - | - | **KY223578** | - |
| Isolate2-27 | HSV-2 | Genital | Yes | 2005 | United States | - | - | **KY223579** | - |
| Isolate2-28 | HSV-2 | Genital | Yes | 2005 | United States | - | - | **KY223580** | - |
| Isolate2-29 | HSV-2 | Genital | Yes | 2003 | United States | - | - | **KY223581** | - |
| Isolate2-31 | HSV-2 | Genital | Yes | 2004 | United States | - | - | **KY223582** | - |
| Isolate2-33 | HSV-2 | Rectal | Yes | 2005 | United States | - | - | **KY223583** | - |
| Isolate2-36 | HSV-2 | Genital | No | 2006 | United States | - | - | **KY223584** | - |
| Isolate2-37 | HSV-2 | Genital | Yes | 2005 | United States | - | - | **KY223585** | - |
| Isolate2-38 | HSV-2 | Rectal | Yes | 2008 | United States | - | - | **KY223586** | - |
| Isolate2-39 | HSV-2 | Genital | Yes | 2006 | United States | - | - | **KY223587** | - |
| Isolate2-41 | HSV-2 | Genital | No | 2008 | United States | - | - | **KY223588** | - |
| Isolate2-42 | HSV-2 | Genital | Yes | 2005 | United States | - | - | **KY223589** | - |
| Isolate2-43 | HSV-2 | Genital | Yes | 2005 | United States | - | - | **KY223590** | - |
| Isolate2-44 | HSV-2 | Genital | Yes | 2005 | United States | - | - | **KY223591** | - |
| Isolate2-45 | HSV-2 | Genital | Yes | 2008 | United States | - | - | **KY223592** | - |
| Isolate2-47 | HSV-2 | Buttock | Yes | 2005 | United States | - | - | **KY223593** | - |
| Isolate2-48 | HSV-2 | Genital | Yes | 2006 | United States | - | - | **KY223594** | - |
| Isolate2-49 | HSV-2 | Genital | Yes | 2005 | United States | - | - | **KY223595** | - |
| Isolate2-50 | HSV-2 | Genital | No | 2008 | United States | - | - | **KY223596** | - |
| Isolate2-53 | HSV-2 | Genital | Yes | 2008 | United States | - | - | **KY223597** | - |
| na, not available.  GenBank accession numbers in bold and underlined represent the new sequences obtained in this research. | | | | | | | | | |
