# Supplementary material for: The herpevac trial for women: Sequence analysis of glycoproteins from viruses obtained from infected subjects
Source: PLoS One. 2017 Apr 27;12(4):e0176687. doi: 10.1371/journal.pone.0176687 (PMC5407825; doi:10.1371/journal.pone.0176687)
Supplement: S2 Table — (DOCX) [file pone.0176687.s002.docx]

| **S2 Table. Ratio of non-synonymous to synonymous evolutionary substitutions (*d*N/*d*S ratio) within the US27 (gB), UL44 (gC), US6 (gD) and US8 (gE) genes for all HSV-1 and -2 strains sampled.** | | | | | | | |
| --- | --- | --- | --- | --- | --- | --- | --- |
| **Glycoprotein B** | | | | | | | |
| **HSV-1** | | | | **HSV-2** | | | |
| **Strain** | **mean *d*N** | **mean *d*S** | ***d*N/*d*S** | **Strain** | **mean *d*N** | **mean *d*S** | ***d*N/*d*S** |
| Sample2 | 0.004 | 0.014 | 0.273 | Sample6 | 0.001 | 0.001 | **1.556*** |
| Sample3 | 0.002 | 0.017 | 0.126 | Sample8 | 0.001 | 0.002 | 0.800 |
| Sample5 | 0.003 | 0.013 | 0.197 | Sample15 | 0.002 | 0.001 | **2.644*** |
| Sample7 | 0.002 | 0.010 | 0.220 | Sample16 | 0.002 | 0.001 | **1.778*** |
| Sample11 | 0.003 | 0.018 | 0.156 | Sample17 | 0.001 | 0.002 | 0.800 |
| Sample20 | 0.003 | 0.012 | 0.231 | Sample18 | 0.002 | 0.002 | 0.848 |
| Isolate1-15 | 0.002 | 0.012 | 0.189 |  |  |  |  |
| Isolate1-16 | 0.002 | 0.012 | 0.182 |  |  |  |  |
| **UL27 gene average obtained in this research** | | | **0.20** | **UL27 gene average obtained in this research** | | | **1.40** |
| **UL27gene average obtained by Lamers et al. (2015)** | | | **0.20** | **UL27 gene average obtained by Lamers et al. (2015)** | | | **0.65** |
| **UL27 gene average obtained by Szpara et al. (2014)** | | | **0.15** | **UL27 gene average obtained by Newman et al. (2015)** | | | **0.67** |
| **Glycoprotein C** | | | | | | | |
| **HSV-1** | | | | **HSV-2** | | | |
| **Strain** | **mean *d*N** | **mean *d*S** | ***d*N/*d*S** | **Strain** | **mean *d*N** | **mean *d*S** | ***d*N/*d*S** |
| Sample2 | 0.006 | 0.012 | 0.460 | Sample6 | 0.002 | 0.007 | 0.215 |
| Sample3 | 0.006 | 0.011 | 0.542 | Sample8 | 0.002 | 0.003 | 0.500 |
| Sample5 | 0.009 | 0.015 | 0.595 | Sample15 | 0.002 | 0.005 | 0.453 |
| Sample7 | 0.006 | 0.010 | 0.556 | Sample16 | 0.006 | 0.003 | **1.968*** |
| Sample11 | 0.009 | 0.017 | 0.514 | Sample17 | 0.002 | 0.003 | 0.500 |
| Sample20 | 0.007 | 0.016 | 0.471 | Sample18 | 0.002 | 0.003 | 0.758 |
| Isolate1-15 | 0.008 | 0.013 | 0.595 |  |  |  |  |
| Isolate1-16 | 0.005 | 0.012 | 0.418 |  |  |  |  |
| **UL44 gene average obtained in this research** | | | **0.52** | **UL44 gene average obtained in this research** | | | **0.73** |
| **UL44 gene average obtained by Lamers et al. (2015)** | | | **0.75** | **UL44 gene average obtained by Lamers et al. (2015)** | | | **0.70** |
| **UL44 gene average obtained by Szpara et al. (2014)** | | | **0.45** | **UL44 gene average obtained by Newman et al. (2015)** | | | **0.80** |
| **Glycoprotein D** | | | | | | | |
| **HSV-1** | | | | **HSV-2** | | | |
| **Strain** | **mean *d*N** | **mean *d*S** | ***d*N/*d*S** | **Strain** | **mean *d*N** | **mean *d*S** | ***d*N/*d*S** |
| Sample1 | 0.002 | 0.009 | 0.185 | Sample6 | <0.001 | 0.001 | 0.418 |
| Sample2 | 0.003 | 0.011 | 0.239 | Sample8 | <0.001 | 0.001 | 0.418 |
| Sample3 | 0.002 | 0.009 | 0.185 | Sample10 | <0.001 | 0.001 | 0.418 |
| Sample4 | 0.001 | 0.009 | 0.165 | Sample13 | 0.001 | 0.003 | 0.157 |
| Sample5 | 0.001 | 0.015 | 0.096 | Sample15 | <0.001 | 0.001 | 0.418 |
| Sample7 | 0.001 | 0.011 | 0.135 | Sample16 | 0.001 | 0.001 | 0.967 |
| Sample9 | 0.001 | 0.011 | 0.135 | Sample17 | <0.001 | 0.001 | 0.418 |
| Sample11 | 0.002 | 0.011 | 0.152 | Sample18 | 0.001 | 0.004 | 0.379 |
| Sample12 | 0.002 | 0.009 | 0.168 | Isolate2-1 | <0.001 | 0.004 | 0.106 |
| Sample20 | 0.002 | 0.011 | 0.134 | Isolate2-2 | 0.001 | 0.001 | **1.057*** |
| Isolate1-1 | 0.002 | 0.009 | 0.185 | Isolate2-3 | <0.001 | 0.001 | 0.418 |
| Isolate1-3 | 0.003 | 0.009 | 0.312 | Isolate2-4 | <0.001 | 0.001 | 0.418 |
| Isolate1-4 | 0.001 | 0.011 | 0.135 | Isolate2-6 | <0.001 | 0.001 | 0.406 |
| Isolate1-6 | 0.002 | 0.016 | 0.156 | Isolate2-8 | <0.001 | 0.001 | 0.418 |
| Isolate1-7 | 0.002 | 0.019 | 0.120 | Isolate2-11 | 0.001 | 0.004 | 0.358 |
| Isolate1-8 | 0.001 | 0.011 | 0.135 | Isolate2-14 | <0.001 | 0.001 | 0.418 |
| Isolate1-9 | 0.001 | 0.008 | 0.161 | Isolate2-15 | 0.002 | 0.003 | 0.460 |
| Isolate1-11 | 0.002 | 0.016 | 0.105 | Isolate2-16 | 0.001 | 0.003 | 0.184 |
| Isolate1-12 | 0.002 | 0.016 | 0.159 | Isolate2-18 | 0.003 | 0.003 | 0.796 |
| Isolate1-13 | 0.002 | 0.019 | 0.127 | Isolate2-20 | 0.001 | 0.001 | **1.298*** |
| Isolate1-14 | 0.003 | 0.009 | 0.312 | Isolate2-21 | <0.001 | 0.001 | 0.418 |
| Isolate1-15 | 0.001 | 0.020 | 0.071 | Isolate2-23 | 0.001 | 0.001 | **1.298*** |
| Isolate1-17 | 0.002 | 0.016 | 0.156 | Isolate2-24 | 0.001 | 0.001 | **1.008*** |
| Isolate1-18 | 0.002 | 0.009 | 0.185 | Isolate2-25 | <0.001 | 0.001 | 0.418 |
| Isolate1-20 | 0.002 | 0.009 | 0.185 | Isolate2-26 | 0.002 | 0.003 | 0.481 |
| Isolate1-22 | 0.002 | 0.009 | 0.185 | Isolate2-27 | 0.001 | 0.001 | **1.057*** |
| Isolate1-24 | 0.002 | 0.009 | 0.173 | Isolate2-28 | <0.001 | 0.004 | 0.106 |
| Isolate1-25 | 0.002 | 0.019 | 0.122 | Isolate2-29 | <0.001 | 0.001 | 0.464 |
| Isolate1-26 | 0.001 | 0.009 | 0.165 | Isolate2-31 | 0.001 | 0.001 | **1.298*** |
| Isolate1-27 | 0.002 | 0.011 | 0.213 | Isolate2-33 | <0.001 | 0.004 | 0.103 |
| Isolate1-28 | 0.002 | 0.029 | 0.080 | Isolate2-36 | <0.001 | 0.001 | 0.418 |
| Isolate1-29 | 0.002 | 0.016 | 0.156 | Isolate2-37 | 0.001 | 0.003 | 0.164 |
| Isolate1-30 | 0.002 | 0.021 | 0.111 | Isolate2-38 | <0.001 | 0.001 | 0.418 |
| Isolate1-31 | 0.001 | 0.009 | 0.165 | Isolate2-39 | <0.001 | 0.001 | 0.418 |
| Isolate1-32 | 0.002 | 0.015 | 0.104 | Isolate2-41 | <0.001 | 0.001 | 0.418 |
| Isolate1-34 | 0.001 | 0.015 | 0.095 | Isolate2-42 | <0.001 | 0.001 | 0.418 |
| Isolate1-35 | 0.001 | 0.011 | 0.135 | Isolate2-43 | 0.001 | 0.001 | 0.965 |
| Isolate1-36 | 0.001 | 0.011 | 0.135 | Isolate2-44 | 0.001 | 0.001 | **1.298*** |
| Isolate1-37 | 0.002 | 0.009 | 0.185 | Isolate2-45 | <0.001 | 0.001 | 0.406 |
|  |  |  |  | Isolate2-47 | <0.001 | 0.008 | 0.059 |
|  |  |  |  | Isolate2-48 | 0.001 | 0.001 | **1.057*** |
|  |  |  |  | Isolate2-49 | 0.001 | 0.001 | 0.976 |
|  |  |  |  | Isolate2-50 | 0.001 | 0.001 | **1.021*** |
|  |  |  |  | Isolate2-53 | 0.001 | 0.001 | **1.046*** |
| **Us6 gene average obtained in this research** | | | **0.16** | **Us6 gene average obtained in this research** | | | **0.59** |
| **Us6 gene average obtained by Lamers et al. (2015)** | | | **0.20** | **Us6 gene average obtained by Lamers et al. (2015)** | | | **0.21** |
| **Us6 gene average obtained by Szpara et al. (2014)** | | | **0.15** | **Us6 gene average obtained by Newman et al. (2015)** | | | **0.25** |
| **Glycoprotein E** | | | | | | | |
| **HSV-1** | | | | **HSV-2** | | | |
| **Strain** | **mean *d*N** | **mean *d*S** | ***d*N/*d*S** | **Strain** | **mean *d*N** | **mean *d*S** | ***d*N/*d*S** |
| Sample2 | 0.004 | 0.024 | 0.166 | Sample6 | 0.001 | 0.001 | **1.058*** |
| Sample3 | 0.003 | 0.018 | 0.158 | Sample8 | 0.001 | 0.002 | 0.629 |
| Sample5 | 0.004 | 0.026 | 0.170 | Sample15 | 0.002 | 0.003 | 0.656 |
| Sample7 | 0.003 | 0.018 | 0.158 | Sample16 | 0.001 | 0.001 | 0.580 |
| Sample11 | 0.004 | 0.027 | 0.163 | Sample17 | 0.001 | 0.002 | 0.345 |
| Sample20 | 0.004 | 0.021 | 0.173 | Sample18 | 0.001 | 0.001 | **1.058*** |
| Isolate1-15 | 0.004 | 0.028 | 0.157 |  |  |  |  |
| Isolate1-16 | 0.004 | 0.023 | 0.150 |  |  |  |  |
| **Us8 gene average obtained in this research** | | | **0.16** | **Us8 gene average obtained in this research** | | | **0.72** |
| **Us8 gene average obtained by Lamers et al. (2015)** | | | **0.15** | **Us8 gene average obtained by Lamers *et al.* (2015)** | | | **0.70** |
| **Us8 gene average obtained by Szpara et al. (2014)** | | | **0.25** | **Us8 gene average obtained by Newman et al. (2015)** | | | **1.00** |
| * Indicates samples with a *d*N/*d*S value > 1, which reflects positive selection pressure. | | | | | | | |
