# Supplementary material for: The herpevac trial for women: Sequence analysis of glycoproteins from viruses obtained from infected subjects
Source: PLoS One. 2017 Apr 27;12(4):e0176687. doi: 10.1371/journal.pone.0176687 (PMC5407825; doi:10.1371/journal.pone.0176687)
Supplement: S3 Table — (DOCX) [file pone.0176687.s003.docx]

| **S3 Table. Amino acid positions under selection for HSV-1 and HSV-2 using five methods of analysis.** | | | | | | | | | | | | |
| --- | --- | --- | --- | --- | --- | --- | --- | --- | --- | --- | --- | --- |
| **Gene Product** | **Codon Position(s)^a^** | | | | | | | | | | | |
|  | **FEL (0.1) ^b^** | | **SLAC (0.1) ^c^** | | **IFEL (0.1) ^b^** | | **MEME (0.1) ^b^** | | **FUBAR (0.9) ^d^** | | **Agreement ^e^** | |
|  | **positively selected** | **negatively selected** | **positively selected** | **negatively selected** | **positively selected** | **negatively selected** | **positively selected** | **negatively selected** | **positively selected** | **negatively selected** | **positively selected** | **negatively selected** |
| **gB HSV-1** | 0 | 47, 48, 80, 92, 226, 238, 267, 306, 322, 343, 365, 371, 407, 464, 465, 505, 643, 660, 704, 732, 736, 738, 796, 811, 844, 854, 872, | 0 | 238, 732, 844, 854 | 59 | 48, 226, 238, 322, 371, 464, 643, 660, 704, 732, 738, 844, 854 | 77 | 0 | 0 | 47, 48, 80, 92, 226, 238, 267, 306, 322, 343, 365, 371, 407, 464, 465, 643, 660, 704, 732, 736, 738, 796, 811, 844, 854, 872 | 0 | **48**, **226**, **238**, **322**, **371**, **464**, **643**, **660**, **704**, **732**, **738**, **844**, **854** |
|  |  |  |  |  |  |  |  |  |  |  |  |  |
| **gB HSV-2** | 0 | 284, 408, 749 | 0 | 749 | 0 | 408, 749 | 0 | 0 | 7, 49 | 284, 408, 749 | 0 | **408**, **749** |
|  |  |  |  |  |  |  |  |  |  |  |  |  |
| **gC HSV-1** | 0 | 59, 131, 212, 314, 327, 332, 352, 382, 389, 418, 430, 455 | 0 | 327 | 132 | 314, 327, 352, 455 | 0 | 0 | 16 | 59, 131, 212, 314, 327, 332, 352, 389, 418, 430, 455 | 0 | **314**, **327**, **352**, **455** |
|  |  |  |  |  |  |  |  |  |  |  |  |  |
| **gC HSV-2** | 0 | 277, 344, 406, 428 | 0 | 344 | 0 | 344 | 0 | 0 | 0 | 277, 344, 406, 428 | 0 | **344** |
|  |  |  |  |  |  |  |  |  |  |  |  |  |
| **gD HSV-1** | 0 | 34, 159, 177, 225, 228, 302, 316, 321, 323, 336, 337, 349, 388 | 0 | 321 | 0 | 34, 159, 177, 228, 321 | 0 | 0 | 0 | 34, 159, 177, 225, 228, 316, 321 | 0 | **34**, **159**, **177**, **228**, **321** |
|  |  |  |  |  |  |  |  |  |  |  |  |  |
| **gD HSV-2** | 0 | 35, 183, 240, 245 | 0 | 0 | 0 | 0 | 0 | 0 | 0 | 35, 183, 240, 245 | 0 | 0 |
|  |  |  |  |  |  |  |  |  |  |  |  |  |
| **gE HSV-1** | 0 | 18, 57, 79, 98, 168, 242, 295, 296, 299, 336, 350, 382, 405, 413, 434 | 0 | 299 | 0 | 18, 299, 382 | 0 | 0 | 0 | 18, 57, 168, 242, 295, 296, 299, 336, 350, 382, 413, 434 | 0 | **18**, **299**, **382**, |
|  |  |  |  |  |  |  |  |  |  |  |  |  |
| **gE HSV-2** | 0 | 471 | 0 | 0 | 0 | 0 | 0 | 0 | 0 | 385, 471 | 0 | 0 |
| ^a^ Codon position starting from the first codon in the reference sequence.  ^b^ The number in parentheses is the cutoff *P* value for the single degree of freedom likelihood ratio test to classify a site as positively or negatively selected.  ^c^ The number in parentheses is the cutoff *P* value for the two-tailed extended binomial test to classify a site as positively or negatively selected.  ^d^ The number in parentheses is the cutoff *P* value for the Bayes factor indicating positive or negative selection at a given site.  ^e^ A least three methods indicated positive or negative selection at the coding position(s) listed. | | | | | | | | | | | | |
